# Electrolytes for High-Safety Lithium-Ion Batteries at Low Temperature: A Review

**DOI:** 10.3390/polym16182661

**Published:** 2024-09-21

**Authors:** Shuhong Yun, Xinghua Liang, Junjie Xi, Leyu Liao, Shuwan Cui, Lihong Chen, Siying Li, Qicheng Hu

**Affiliations:** 1 Guangxi Key Laboratory of Automobile Components and Vehicle Technology, Guangxi University of Science and Technology, Liuzhou 545006, China; 2 Industry College of Intelligent Vehicle (Manufacturing) and New Energy Automobile, Guangxi University of Science and Technology, Liuzhou 545006, China; 3 Zhejiang Kaili New Materials Co., Ltd., Shaoxing 312000, China

**Keywords:** lithium-ion batteries, low temperatures, safety issues, solid-state electrolytes

## Abstract

As the core of modern energy technology, lithium-ion batteries (LIBs) have been widely integrated into many key areas, especially in the automotive industry, particularly represented by electric vehicles (EVs). The spread of LIBs has contributed to the sustainable development of societies, especially in the promotion of green transportation. However, the high demand for battery performance and safety in these fields has made the high viscosity, volatility, and potential leakage inherent in traditional organic liquid electrolytes a constraint on their further expansion. Especially at low temperature, the increased viscosity of the electrolyte, reduced solubility of lithium salts, crystallization or solidification of the electrolyte, increased resistance to charge transfer due to interfacial by-products, and short-circuiting due to the growth of anode lithium dendrites all affect the performance and safety of LIBs. Therefore, improving the safety performance of LIBs under low-temperature environments has become a focus of current research. This paper primarily reviews the progress made in utilizing different types of electrolytes in LIBs to enhance safety and optimize low temperature performance and discusses the current research progress as well as the future development direction of the field.

## 1. Introduction

With the development of technology and the increasing demand for energy, lithium-ion batteries (LIBs) have become the mainstream battery type due to their high energy density, long lifespan, and light weight [1,2]. As electric vehicles (EVs) continue to revolutionize transportation, their ability to operate reliably in extreme conditions, including subzero temperatures, becomes critical. At present, EVs and many high-tech fields, such as aerospace, polar research, and military equipment, often need to operate in extremely low-temperature environments [3,4]. The challenges of capacity decay and charge–discharge inefficiency in subzero environments limit the wider application of LIBs [5]. The ion transference at the interface is hindered at low temperature (LT), causing high interface impedance and high interface polarization. These problems greatly affect the performance of the battery, resulting in longer charging times, shorter cycle life, lower battery capacity, faster decay rate, and worse rate capability [4,6,7,8].

The material of the electrode, electrolyte, and separator, and the structure of the battery all affect the working performance of LIBs at LT [9,10]. As a key component of LIBs, the electrolyte is known as the “blood” of the battery, and is a compound that can conduct electricity by dissociating into free solvent molecules, which move directionally under the influence of an electric field, thereby forming an electric current. Traditional LIBs’ electrolyte is mainly composed of organic solvents, conductive lithium salts, and additives formulated in proportion. These components together determine the electrochemical performance, thermal stability, and safety of the electrolyte [11].

Liquid electrolytes using high melting point solvents become more viscous or even solidify at LTs. The viscosity of the electrolyte increases, affecting the wettability of the electrolyte on the electrode surface. The migration rate of Li^+^ in the electrolyte decreases, which leads to a significant decrease in ionic conductivity [12,13]. The solid electrolyte interface (SEI) formed between the electrolyte and the electrode is unevenly distributed, leading to an increase in the interfacial impedance, which is not conducive to the stable cycling of the battery [14,15]. Studies have shown that at temperatures below −20 °C, the reversible capacity of LIBs drops to 25% or less of that at room temperature (RT), and the lost capacity can usually be recovered as the temperature rises [15]. If the battery is used in extremely cold environments, such as regions above 30 degrees north and south latitude or the outer layer of Mars, the battery will reach the discharge cut-off voltage faster. Part of the battery capacity cannot be discharged in the normal voltage range and the battery capacity decreases irreversibly [15]; when the temperature recovers, the battery capacity cannot be restored [16]. At extremely LTs, the viscosity of the electrolyte increases significantly, leading to a significant decrease in the migration rate of Li^+^ within the electrolyte. Li^+^ is unable to intercalate and deintercalated ions in a timely manner, and directly obtains electrons on the surface of the anode to become lithium metal (Li plating phenomenon). Although the viscosity of the electrolyte decreases after the temperature is restored, the precipitated lithium metal cannot be completely re-intercalated into the anode during the discharge process. This portion of lithium becomes “dead lithium”, leading to an irreversible loss of capacity. The lithium metal precipitated on the anode surface reacts with the electrolyte, and the deposition of the reaction product thickens the solid electrolyte interface layer (SEI), which increases the internal resistance of the battery and results in an irreversible loss of Li^+^. Additionally, the process of lithium precipitation consumes Li^+^, further diminishing the battery’s capacity [17].

The direct consequence of LT is a reduction in battery capacity and a decrease in discharge capability, but changes in the internal material features of the battery at LT can also be the trigger for triggering thermal runaway. The term “battery thermal runaway” is used when the active material of the battery undergoes a violent exothermic reaction under the influence of various factors, which makes the internal temperature of the battery rise out of control [18,19].

**Figure 1:**
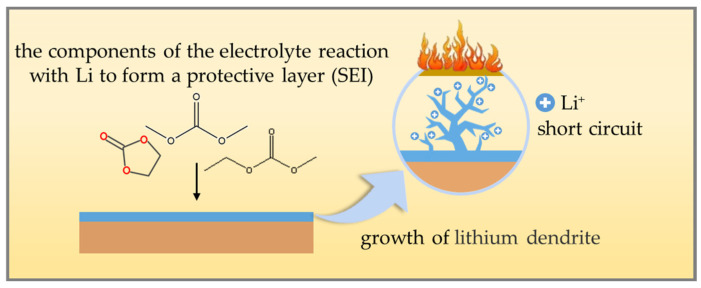
Schematic diagram of the growth of lithium dendrite.

Low-temperature thermal runaway often occurs during rapid charging and discharging [20]. This is because the low temperature limits the diffusion rate of ions and Li^+^ cannot be sufficiently intercalated in the electrode material [21], leading to the formation of lithium dendrites on the surface of the anode (as shown in Figure 1). The generation of lithium dendrites further aggravates the charge accumulation on the surface of the anode, leading to an imbalance in the battery voltage and exacerbating the polarization phenomenon inside the battery [22]. With the continuous growth of lithium dendrites, they may eventually puncture the internal diaphragm of the battery, leading to direct contact between the electrodes and an internal short circuit. The short circuit will rapidly generate a large amount of heat, causing a dramatic increase in battery temperature and triggering a thermal runaway of the battery [23,24,25].

In the field of LIBs, the performance optimization of electrolytes has become a decisive factor in overcoming the dual challenges of performance degradation and an increased risk of thermal runaway at LT. Many scholars have reviewed the development of low-temperature electrolytes or high-safety electrolytes. However, in the application of LIBs, it is essential to consider both the low-temperature performance and the high safety of the batteries. In this paper, we review the methods and strategies for improving the low-temperature performance and safety properties of organic liquid electrolytes, polymer/solid-state electrolytes, ionic liquid-based electrolytes, and inorganic liquid electrolytes. The study is expected to provide a valuable reference for future battery device applications in LT extreme environments.

## 2. Organic Liquid Electrolytes

Organic liquid electrolytes are electrolyte systems formed by dissolving lithium salts in one or a mixture of strongly polar organic solvents. In a LT environment, the solvent used in organic electrolyte crystallizes, which affects the performance of the battery. To improve the performance of organic liquid electrolytes at LT, low melting point co-solvents, the mixing of different types of lithium salts, and reasonable control of salt concentration are usually used [26].

### 2.1. Co-Solvents with Low Melting Point

Commonly used organic electrolyte solvents include ethylene carbonate (EC), diethyl carbonate (DEC), dimethyl carbonate (DMC), and ethyl methyl carbonate (EMC). Among these, EC is a crucial cyclic carbonate with a high potential for film formation on the anode [27]. It can preferentially precipitate during the charging process and participate in the formation of the SEI layer. The resulting SEI layer mitigates direct contact between the electrolyte and the anode, thereby slowing down the decomposition of the electrolyte and corrosion of the anode. However, EC has a high melting point (36.4 °C), making it solid at RT and resulting in poor low-temperature performance. By mixing low melting point organic solvents, the interaction between different solvent molecules can reduce the overall solidification point of the electrolyte. In practice, it is often blended with other linear carbonate solvents with lower melting points, such as DEC (−43 °C), DMC (4.6 °C), and EMC (−53 °C).

**Figure 2:**
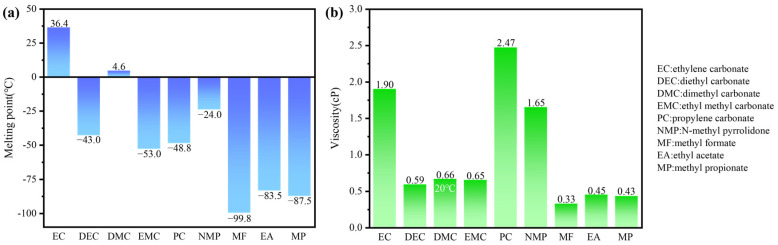
Comparison of melting points (**a**) and viscosity (**b**) of common organic solvents at 25 °C.

Both propylene carbonate (PC) and EC are carbonates with similar carbonate groups (CO_3_^2−^). PC, characterized by its low melting point (−48.8 °C) [28] and low viscosity (2.47 cP), can serve as an excellent solvent alternative for low temperature electrolytes. The melting points and viscosities of common organic solvents, including EC, DEC, DMC, EMC, PC, NMP (N-methyl pyrrolidone), MF (methyl formate), EA (ethyl acetate), and MP (methyl propionate), at 25 °C are shown in Figure 2.

To investigate the effects of PC on LIBs at LT, Zhang et al. [5] assembled the batteries with 1 M LiPF_6_ in EC/EMC (3:7 by mass) and 1 M LiPF_6_ in PC/EC/EMC (1:1:3 by mass), respectively, and carried out the low-temperature constant-current cycling test. The results showed that the operating voltage and capacity of the batteries decreased with decreasing temperatures. At −20 °C, the capacity retention of the battery with PC was 83%, while the capacity retention of the battery without PC was only 70%. The addition of PC reduces the viscosity of the electrolyte, resulting in better wetting of the electrode surface at low temperatures and reduced interfacial resistance. As a result, Li^+^ is easier to migrate and transport, thereby increasing capacity. This shows that the addition of PC can significantly improve the LT capacity of LIB.

**Figure 3:**
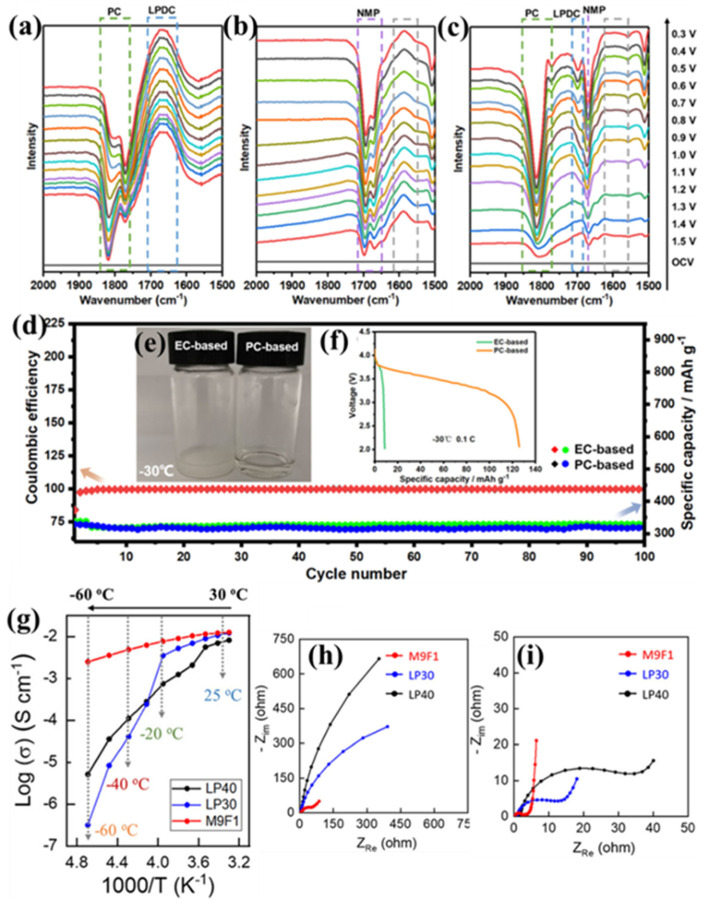
(**a**–**c**) In situ FTIR spectra of different electrolytes: 1 M LiPF_6_ in PC (**a**), 1 M LiPF_6_ in NMP (**b**), and 1 M LiPF_6_ in PC/NMP (2:1 wt.%) (**c**). (**d**) Cyclic performance (0.2 C) of Li//graphite half cells with EC-based and PC-based electrolytes at RT. (**e**) Optical photos of both electrolytes at −30 °C. (**f**) Discharge curves of LiNi_0_._8_Co_0_._1_Mn_0_._1_O_2_//graphite full-cell at −30 °C. (Reprinted with permission from [30]; copyright 2022, ACS Applied Materials & Interfaces). (**g**) Ionic conductivities of different electrolytes measured at various temperatures from −60 to 30 °C. Nyquist plots of the batteries after rate capabilities at the different temperature between (**h**) −20 °C and (**i**) room-temperature. (Reprinted with permission from [34]; copyright 2021, ACS Energy Letters).

However, the interface problem between PC and the graphite anode has been limiting for its further application. The carbonate dibasic anion (CO_3_^2−^) in PC is easily adsorbed by the oxygen atoms on the surface of the graphite anode for bonding reactions, which accelerates the aging of the graphite anode material [29]. Zhang et al. [30] discovered that the solvent co-intercalating disappeared by optimizing the volume ratio of PC and NMP in the EC-free electrolyte (1 M LiPF_6_ in PC/NMP (2:1 by mass)), which led to the reversible intercalation of Li^+^ into graphite. In situ Fourier transform infrared spectroscopy (FTIR) studies (Figure 3a–c) confirmed the ability of NMP to mitigate the reduction in PC at the graphite-electrolyte interface. Long-cycle tests on Li//graphite half-cells at 0.2 C under RT conditions revealed that the PC-based electrolyte was well compatible with graphite, and its cycling performance was comparable to that of the EC-based electrolyte (Figure 3d). Furthermore, the freezing point of the PC-based electrolyte at −30 °C is significantly lower than that of the commercial EC-based electrolyte (Figure 3e), so the synergistic effect of NMP and PC contributes to the enhancement of battery performance at LTs. The LiNi_0.8_Co_0.1_Mn_0.1_O_2_//graphite full-cell using a commercial electrolyte could not be discharged at −30 °C, whereas the battery employing the optimized PC-based electrolyte (1 M LiPF_6_ in PC/NMP (2:1 by mass) could deliver a discharge capacity of 125.9 mAh g^−1^, which is approximately 65% of the capacity at RT (Figure 3f).

Apart from carbonates, esters and their cyclic derivatives (called lactones) can also be used as electrolyte solvents in low-temperature LIBs. Esters have the advantages of low viscosity, a low melting point, and moderate polarity, all of which promote rapid Li^+^ transfer over a wide temperature range. However, they have several distinct drawbacks over carbonates, such as being more flammable and having a narrower electrochemical window. The simplest esters, such as MF [31] and EA [32], are unstable in lithiated graphite and do not form a passivation layer on the anode upon decomposition. MP is particularly promising for low-temperature electrolytes because of its low melting point of −87.5 °C and low viscosity (0.43 cP), which represents the lowest viscosity of the conventional carbonate solvent family [33].

These physical properties of MP lead to high ionic conductivity at LTs. Cho et al. [34] compared the ester-based M9F1 electrolyte (1 M LiPF_6_ in MP: fluorinated ethylene carbonate (FEC) (90:10 by volume) with the conventional EC-based LP40 electrolyte (1 M LiPF_6_ in EC/DEC (5:5 by volume) and LP30 electrolyte (1 M LiPF_6_ in EC/DMC (5:5 by volume) for comparison. The ionic conductivity of M9F1 is superior to that of both LP40 and LP30 from −60 to 30 °C, especially below −20 °C (Figure 3g). Applying M9F1 to the LiNiMnCoO_2_//graphite pouch full-cell, charged at 0.1 C at RT and discharged at −20 °C, the discharge capacity maintained 84% of the RT capacity (133 mA h g^−1^). Meanwhile, the LP30 and LP40 maintained 61% and 33%, respectively. The full battery was further investigated via electrochemical impedance spectroscopy (EIS) (Figure 3h–i). The cathode resistance of LP30 and LP40 was found to increase significantly at −20 °C compared to RT, indicating that the charge transfer resistance (R_ct_) on the cathode is more limited in this temperature range. In contrast, the M9F1 electrolyte has an extremely low cathode R_ct_ at −20 °C, suggesting that it is an excellent electrolyte for enhancing the low-temperature cycling performance of batteries.

These studies have shown that the overall viscosity of the electrolyte in LT can be effectively decreased by adding or replacing co-solvents with low melting points and changing the mixing ratios. This directly leads to a reduction in the transfer resistance of ions in the electrolyte, further enhancing the coulombic efficiency of the battery and reducing the internal resistance. This further suggests that the low-temperature physical nature of the organic solvents used in conventional organic liquid electrolytes is important. However, no research was found regarding the safety of low melting point co-solvents at LT.

### 2.2. LiBF_4_ and LiODFB Mixed-Salt Electrolyte

Although lithium salts are often used as electrolyte additives, few studies have been conducted to investigate their role under LT conditions. The impedance spectra of lithium-electron batteries with LiBF_4_ and LiPF_6_ as the electrolyte salts were measured under fully charged and fully discharged states at −20 °C. [15] The results show that in both states, the R_ct_ of LiBF_4_ is much smaller than that of LiPF_6_. Therefore, it is speculated that LiBF_4_-based electrolytes have a small charge transfer resistance, which may improve the low-temperature performance of LIBs [15,35].

Ding et al. [36] demonstrated that the ionic conductivity of lithium salts depends on the dissociation degree of the lithium salt and the viscosity of the solution, which mutually govern each other. The molecular structure of lithium difluoro (oxalate)borate (LiODFB) contains Li^+^, oxalate ions (C_2_O_4_^2−^) and a borate ion formed by two fluorine atoms and two boron atoms (BF_2_O_2_^−^). The anionic portion of LiODFB encompasses multiple oxygen atoms, which form hydrogen bonds with solvent molecules to enhance solubility, resulting in lower viscosity and superior LT performance of electrolytes containing LiODFB [37]. Therefore, many researchers applied both LiBF_4_ and LiDFOB as additives to improve the low-temperature properties [38,39,40,41].

**Figure 4:**
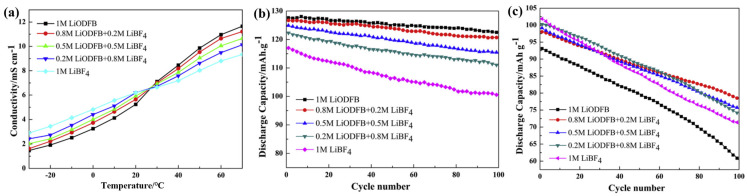
(**a**) Conductivity of different electrolyte systems at different temperatures. (**b**,**c**) Cycling curves of the LiNi_0.5_Mn_1.5_O_4_//graphite cells with different electrolyte systems at different temperatures: 25 °C (**b**) and −20 °C (**c**). (Reprinted with permission from [38]; copyright 2016, Journal of Power Sources).

Zhang et al. [41] analyzed the performance of the mixed-salt electrolyte (LiBF_4_ and/or LiODFB in EC: PC: EMC (1:1:1 by mass) in the range of −40~60 °C. The ionic conductivity of the mixed-salt electrolyte is always better than that of the single-salt electrolyte at all temperatures. Zhou et al. [38] studied the performance of the mixed-salt electrolyte (EC/DMC/EMC (1:1:1 by mass) of LiBF_4_ and LiODFB at −30~70 °C. Consistent with the results of Zhang et al., the ionic conductivity of the mixed-salt electrolyte is consistently better than that of the single-salt electrolyte at different temperatures (Figure 4a). Different proportions of mixed-salt electrolytes were applied to the LiNi_0.5_Mn_1.5_O_4_//graphite full-cell, and it was found that the mixed salts could improve the cycle performance of the single-salt electrolyte LiNi_0_._5_Mn_1_._5_O_4_//graphite full-cell at 25 °C and −20 °C (Figure 4b,c). Li et al. dissolved 0.9 M LiODFB/LiBF_4_ (0.037:0.011 by mole ratio) of mixed salt in EC/DMS/EMC mixed solvent (1:1:3 by volume) and tested its electrochemical performance [42]. It was shown that after 50 cycles of LiFePO_4_/Li half batteries with different electrolytes with a discharge rate of 0.5 C at 20 °C, batteries with both LiODFB/LiBF_4_-based electrolytes showed higher capacity retention (89.25%) than those with LiPF_6_-EC/DEC/DMC/EMC electrolytes (88.49%). In addition, the LiODFB/LiBF_4_-based electrolytes showed better capacity retention (94.57%) after 50 cycles with 0.5 C at −20 °C. As a result, the superior cycle stability of LiODFB/LiBF_4_ mixed-salt electrolytes over single-salt electrolytes was demonstrated.

By comparing the electrochemical tests of different ratios of LiODFB and LiBF_4_ mixed salts in different solvents, it was found that the cycling stability and ionic conductivity of the battery under low-temperature conditions were relatively improved. Different lithium salts exhibit varying solvation abilities in solvents. LiODFB has a greater anionic radius and less ionic association in solution, which helps to improve the conductivity of the electrolyte and thus improves the performance of the battery at low temperatures. LT conditions will thicken the resulting SEI layer during the first graphite-lithiation process. The SEI layer formed by LiODFB/LiBF_4_ electrolytes is more effective in inhibiting the decomposition of the electrolyte than that formed by LiPF_6_-based electrolytes, resulting in a thinner SEI layer. This indicates that the mixing of lithium salts with different roles can improve the LT performance of the battery under certain conditions. However, safety related studies of organic electrolytes with mixed salts have not been presented.

### 2.3. High-Concentration Electrolytes

Commercial LIBs consist of conductive lithium salts dissolved in an organic solvent at a concentration of about 1 mol L^−1^ (1 M) [43,44]. In conventional organic solvents, when lithium salts are mixed with a very small amount of solvent to form a very concentrated solution, all the solvent molecules are coordinated to the cation and still maintain their fluidity [45]. When the salt concentration in electrolytes reaches a certain level, the chemical structure of the solution changes dramatically. The concentration of free solvent molecules and solvent-separated ion pairs decreases, while the concentration of contact ion pairs and aggregates increases, and this electrolyte is referred to as a high-concentration electrolyte [46,47,48].

Compared to traditional electrolytes, high-concentration electrolytes have superior physical properties, such as a higher Li^+^ transference number [33,49]. The salt content in highly concentrated electrolytes is several times higher than in conventional electrolytes, so the ratio of free solvent molecules is relatively small [50,51,52]. In highly concentrated electrolyte solutions, the solvent molecules are immobilized by a high salt concentration, which prevents them from evaporating and catching fire, resulting in higher thermal stability. Numerous studies [50,53] have been conducted to evaluate the superior nonflammability of lithium salt electrolytes at high concentrations. Nevertheless, because of the high salt concentration in the organic electrolyte, viscosity will increase at LTs. To address this issue, additives are frequently added to the electrolyte for dilution [54].

**Figure 5:**
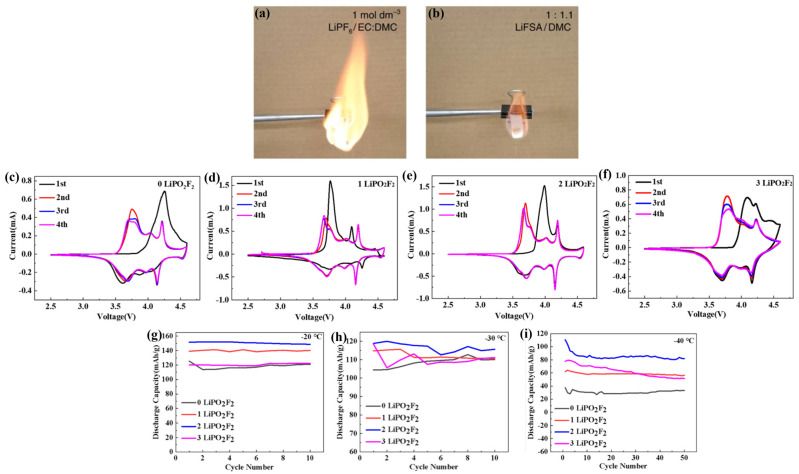
(**a**,**b**) Burning test: (**a**) 1 M LiPF_6_ EC/DMC, (**b**) LiFSA/DMC. (Reprinted with permission from [50]; copyright 2016, Nature Communications) (**c**–**f**). Different LiPO_2_F_2_ contents’ cyclic voltametric curves between 2.75 and 4.2 V: (**a**) 0 wt.% LiPO_2_F_2_; (**b**) 1 wt.%; LiPO_2_F_2_; (**c**) 2 wt.% LiPO_2_F_2_; and (**d**) 3 wt.% LiPO_2_F_2_. (**g**–**i**) Batteries’ cycling curves at (**e**) −20 °C, (**f**) −30 °C, and (**g**) −40 °C. (Reprinted with permission from [62]; copyright 2022, ACS Applied Energy Materials).

Wang et al. [50] investigated the effects of different concentrations of lithium bis(fluorosulfonyl)amide (LiFSA) salts in different ratios of DMC and EC mixed solvents. Irrespective of the solvent used, the viscosity of the electrolyte increased with an increasing LiFSA molar fraction (X_LiFSA_). When the X_LiFSA_ was higher than 0.14, solutions with DMC as the single solvent showed higher ionic conductivity than mixed solutions of EC and DMC due to the low viscosity of DMC at high concentrations. This result suggests that viscosity becomes a decisive factor for the ionic conductivity of concentrated solutions. Interestingly, the burning test (Figure 5a,b) showed that the concentrated electrolyte (LiFSA/DMC 1:1.1 by volume) burned less violently compared to the commercial electrolyte (LiPF_6_/DMC 1:1 by volume), demonstrating that the concentrated electrolyte is significantly safer due to its superior thermal stability and flame-retardant capabilities. Zhang et al. [54] obtained relatively high ionic conductivity by dissolving 2.4 M lithium bis(fluorosulfonyl)imide (LiFSI) in a mixed solvent of fluorinated vinyl carbonate (FEC) and DMC (FEC/DMC 3:7 by volume). No observable capacity degradation was observed in the Li//LiFePO_4_ half-cell over 2600 long-term cycles at 0 °C at a rate of 1 C, with an average CE of 99.6% over 400 cycles at −20 °C at a rate of 0.1 C.

High-concentration lithium salt electrolytes significantly enhance the flame retardancy of traditional electrolytes, effectively improving the safety of the battery. The utilization of dual-salt/mixed-salt electrolytes, based on this high single-salt concentration, further optimizes the electrolyte’s performance through the synergistic effect of the different lithium salts [55,56,57,58,59]. Lin et al. [60] dissolved lithium bis (trifluoromethylsulphonyl)imide (LiTFSI) in a mixed solvent of cyclobutene sulfone (TMS) and ethyl acetate (EA) (TMS/EA 3:7 by volume) to prepare the electrolytes (TE-xm-LiTFSI, x = 1~6). At 25 °C, the viscosity of the TE-xm-LiTFSI increased with the addition of LiTFSI (1~6 M), and the ionic conductivity was negatively correlated with the viscosity. Based on the results of the viscosity and ionic conductivity of TE-xm-LiTFSI, the molar concentration of the Li salt was fixed at 4 M, and LiTFSI was partially replaced by lithium difluoro(oxalato)borate (LiDFOB) to observe the performance of the dual-salt high-concentration electrolyte (TE-4m-LiTD). At 25 °C, as the amount of added LiDFOB increased, the ionic conductivity gradually increased, reaching a peak for TE-4m-LiTD (n_LiDFOB_:n_LiTFSI_ = 4.3:1) before slowly decreasing, while the viscosity reached a minimum and then increased. Moreover, after adding hydrofluoroether (HFE) diluent to the dual-salt high-concentration electrolyte (TEH-2m-LiTD with 10 wt.%HFE), it remains liquid even at −80 °C, thus allowing the Li//NCM523 half-cell to provide 75% of the RT capacity (102 mAh g^−1^/136 mAh g^−1^) under the conditions of 0.1 C at −40 °C, showing excellent low-temperature performance.

Zhao et al. [61] dissolved LiTFSI and LiDFOB in trimethyl phosphate (TMP, melting point of −46 °C) and/or γ-butyrolactone (GBL, melting point of −44 °C) to formulate a dual-salt electrolyte. The NCM622//mesocarbon microbeads (MCMB) full-cell at 0.5 C using 2 M LiTFSI + 2 M LiDFOB-TMP/GBL exhibits the best cycling performance compared to the single salt (4 M LiTFSI-TMP/GBL or 4 M LiDFOB-TMP/GBL) in a RT environment, showing 83.8% capacity retention (131.7 mAh g^−1^/157.2 mAh g^−1^) and a high average coulombic efficiency of 99.5%. The capacity retentions with 2 M LiTFSI + 2 M LiDFOB-TMP/GBL were 83.2% (135.7 mAh g^−1^/163.1 mAh g^−1^), 77.1% (125.9 mAh g^−1^/163.3 mAh g^−1^), and 44.8% (73.7 mAh g^−1^/164.7 mAh g^−1^) at −10 °C, −20 °C, and −30 °C, respectively, when charging at 0.1 C at RT.

Li et al. [62] analyzed the effect of difluoro phosphate (LiPO_2_F_2_) concentration on the performance of electrolytes (1 M LiPF_6_ DMC/EMC/PC/FEC). The cyclic voltametric curves of the NCM811//Li half-cell containing different concentrations of LiPO_2_F_2_ were tested at RT (Figure 5c–f). The cyclic voltametric curves of the electrolytes with 1 wt.% (Figure 5d) and 2 wt.% LiPO_2_F_2_ (Figure 5e) overlapped better in the last three circles compared to the other two electrolytes (Figure 5c,f), indicating better cycling stability. As can be seen in Figure 5g–i, the addition of a small amount of LiPO_2_F_2_ leads to a significant increase in the discharge specific capacity at LT from −20 °C to −40 °C. This is consistent with the previous cyclic voltammetry results.

It is found that the cycle performance and reversibility of electrolytes are improved at LT by using suitable lithium salts and low-melting-point solvents, as well as high concentrations of single-salts/mixed-salts. In high-concentration electrolytes, the increased concentration of salts leads to more complex interactions between solvent molecules and salt ions. The relative reduction in the number of solvent molecules, coupled with the need for each solvent molecule to solvate multiple salt ions, promotes the formation of a tighter and more stable solvation structure. The decrease in free space for solvent molecule movement and the reduced volatility of the solvent directly lowers the flammability of the electrolyte. Additionally, the stable solvation structure also contributes to reducing direct contact between lithium salt ions, thereby mitigating ion aggregation and enhancing the ionic mobility and conductivity of the electrolyte.

In summary, by integrating strategies such as incorporating low-melting-point co-solvents, blending mixed lithium salts, and adopting high-concentration salt electrolytes, we can effectively mitigate the challenges posed by the decline in ionic conductivity of the electrolyte, the increase in viscosity, and lithium dendrite formation at LTs. Notably, the high-concentration lithium salt electrolyte, owing to its unique solvation structure, exhibits flame-retardant properties, which can help prevent battery fires or explosions under extreme conditions like short circuits or overcharges, thereby enhancing battery safety. In conclusion, these electrolyte strategies pave the way for new avenues in the low-temperature applications of LIBs.

## 3. Deep Eutectic Solvents-Based Electrolytes (DEEs)

To address the uncontrollable growth of lithium dendrites induced by carbonate-based electrolytes in LIBs and the serious related safety issues, some researchers have proposed a new electrolyte based on deep eutectic solvents (DESs) [63] with a view to ameliorating these challenges. DESs are a type of solvent with a low melting point, consisting of two or more substances (usually a solid hydrogen bond acceptor (HBA) and a solid hydrogen bond donor (HBD)) mixed in a certain ratio [64]. This mixture forms a eutectic structure through strong interactions between the components (hydrogen bonding, Lewis acid-base interactions, van der Waals forces, etc.), resulting in a melting point that is significantly lower than that of a single component [65]. This special eutectic structure possesses strong intermolecular interaction forces, resulting in low volatility of the solvent and thus ensuring high safety.

Geiculescu et al. [66] reported a class of binary DEEs. These DEEs consist of methanesulfonamide (MSA) or N,N-dimethylmethanesulfonamide (DMMSA) mixed with LiFSI or LiTFSI, leading to a eutectic phenomenon, whereby the mixtures remain liquid at RT or even −40 °C. With LiTFSI salt, DMMSA/LiTFSI solutions demonstrate consistently higher conductivities than MSA/LiTFSI ones under test conditions ranging from −40 °C to 100 °C. These differences in conductivity increase as the temperature decreases. This is due to the weaker hydrogen bonding ability of DMMSA compared to MSA, which results in a lower viscosity of the DMMSA/LiTFSI solution. On the other hand, the LiFSI-based DEEs (MSA:LiFSI 3:1 and DMMSA:LiFSI 4:1) exhibited higher ionic conductivity than the LiTFSI salt-based DEEs at all temperatures tested, demonstrating an ionic conductivity >1.0 × 10^−4^ S cm^−1^ at −30 °C. This is attributed to the fact that LiFSI dissociates more readily into free solvent molecules in solution compared to LiTFSI, thereby increasing the number of charge carriers.

Two types of DEEs (LiPF_6_-DEE and LiTFSI-DEE) based on methyl carbamate and lithium salts (LiPF_6_ or LiTFSI) were prepared by Hardir et al. [67]. The ionic conductivities of LiPF_6_-DEE and LiTFSI-DEE were 7.78 × 10^−4^ and 8.26 × 10^−4^ S cm^−1^, respectively, under the test conditions at 0 °C. Further, the viscosities of these two DEEs were measured at different test temperatures. The results showed that at 5 °C, the viscosity of LiPF_6_-DEE was about 108 cP, while the viscosity of LiTFSI-DEE was relatively low, at about 95 cP. This is because LiTFSI exhibits better solubility in organic solvents and, therefore, the solvation structure formed by LiTFSI with the methyl carbamate solvent molecules is more stable, which helps to reduce intermolecular resistance and viscosity. This property has positive implications for enhancing the LT performance of batteries.

**Figure 6:**
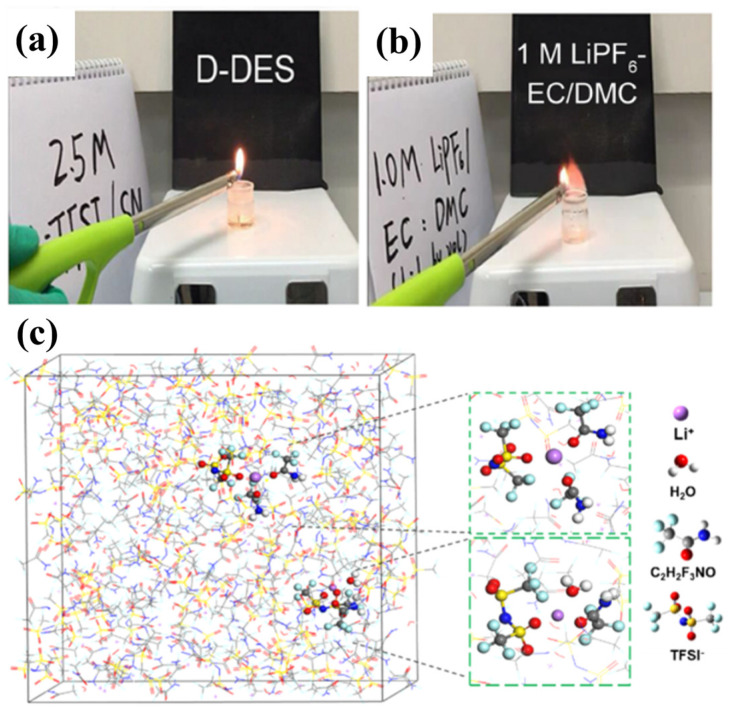
(**a**,**b**) Burning test on a heating plate at 200 °C: (**a**) D-DES, (**b**) 1 M LiPF_6_-EC/DMC. (Reprinted with permission from [68]; copyright 2020, Chemistry of Materials) (**c**) 3D snapshot of LT-1 electrolyte system obtained from MD simulations and partial enlarged snapshot representing Li^+^ solvation structure. (Reprinted with permission from [69]; copyright 2022, Journal of Power Sources).

Hu et al. [68] proposed a non-flammable electrolyte based on a double anion deep eutectic solvent (D-DES), which is a combination of nitrile compounds (succinonitrile, SN) and Li salts (LiTFSI and LiDFOB). Because of its high polarity, SN facilitates the dissociation of Li salts. This D-DES electrolyte demonstrates excellent ionic conductivity (~1.0 × 10^−3^ S cm^−1^ at RT). Flammability tests were performed on both D-DES and a commercial electrolyte (1 MLiPF_6_-EC/DMC), and neither the liquid D-DES nor its surface vapors could be ignited by a cigarette lighter under the test conditions (Figure 6a). LiCoO_2_//D-DES//Li half-cells exhibited excellent electrochemical performance. Under RT conditions, the cells were able to be tested for 1000 cycles at 1 C at a cut-off voltage of 3.0–4.5 V (capacity retention of 80%). This excellent performance may be attributed to the high-voltage resistance property of SN and the film-forming effect of lithium salts, as well as to the intermolecular interactions (coordinative and hydrogen-bonding interactions) in the D-DES that transform the solid component into the liquid phase.

Li et al. [69] prepared eutectic solvent electrolytes (X_LiTFSI_:X_TFA_ molar ratio of 1:2.8) based on a eutectic mixture (referred as LT in [69]) of LiTFSI and trifluoroacetamide (TFA). The eutectic solvents exhibited liquid-state properties due to the strong interaction of amide groups with TFSI^−^. Molecular dynamics simulation (MD) studies show that the addition of a small amount of water (at a low molar ratio) to the eutectic solvent system hydrates Li^+^ (Figure 6c) and increases the electrolyte conductivity by about five times. Therefore, different amounts of water (labeled LT-0.5, LT-1, and LT-2, respectively) were added to eutectic solvents consisting of LiTFSI and TFA for performance testing (adding 0.5, 1, and 2 mol water to the above solution, respectively). After testing, the melting point of the eutectic electrolyte was −55.3 °C, which was further lowered to below −61.6 °C by adding appropriate amounts of water. This allows the cell to exhibit good performance at −20 °C. Lithium Manganate (LiMn_2_O_4_)//LT-1//LiTi_2_(PO_4_)_3_ (titanium phosphate lithium) full-cell exhibits good cycle performance (0 °C~100 mAh g^−1^, −10 °C~95 mAh g^−1^, −20 °C~60 mAh g^−1^) at 0.1 A g^−1^.

Eutectogels (ETGs) obtained by polymerizing monomers dissolved in a DES are potential candidate electrolytes for quasi-solid LIBs due to their low volatility and non-flammable characteristics [70,71]. Combining the small size of water molecules and their miscibility with DESs, Hou et al. [72] introduced a small amount of water into ETGs to prepare new “water-in-eutectogel” (WiETG) electrolytes. WiETG electrolytes were obtained by mixing a carboxymethyl cellulose (CMC)-cross-linked polyacrylamide (PAM) polymer with DESs with different water contents (n_LiTFSI_: n_acetamide_: n_H2O_ = 1:3:x, x = 0, 0.5, 1, and 2, denoted as ACE0, ACE0.5, ACE1, and ACE2, respectively). The WiETG electrolytes obtained by mixing the dried polymers with ACE1 or ACE2 are denoted as CP-131 and CP-132, where CP-131 has a membrane-to-electrolyte weight ratio of 4.7:72.3, and CP-132 has a ratio of 5:79.7. This electrolyte not only retains the advantages of ETGs, but also further enhances the ionic conductivity and electrochemical stability of the electrolyte by adjusting the water content. This enhancement of ionic conductivity is attributed to the formation of strong hydrogen bonds between the water molecules and the hydrophilic groups (-CONH_2_ and -OH, etc.) on the polymer chains, which participate in the Li^+^ solvation structure and achieve faster transport of Li^+^. The LMO//CP-131//LTO pouch cells were tested for cycling at 0 °C, −10 °C, and −20 °C, resulting in discharge capacities of 120 mAh g^−1^, 80 mAh g^−1^, and 40 mAh g^−1^at 1 C, respectively.

In summary, due to the deep eutectic phenomenon, DEEs typically have a lower vapor pressure and higher thermal decomposition temperature, which reduces the risk of volatilization and combustion and improves the safety of LIBs. Its low-temperature performance is improved by the addition of solvents with different low melting points; however, the interaction between solvent molecules and lithium ions in DEEs may form a specific solvent structure, leading to an increase in the interfacial resistance, which affects the battery performance. The current research on DEEs has yet to reach a comprehensive level, so studies focusing on their application in LIBs remain relatively scarce.

## 4. Solid-State Electrolytes (SSEs)

Although organic solvent-based liquid electrolytes have the advantages of high electrical conductivity and superior electrode surface wettability, they also suffer from insufficient electrochemical stability, low ionic selectivity, and low thermal stability [73], especially in the case of overcharging or internal short-circuiting, which can lead to thermal runaway, resulting in safety problems such as smoke, fire, and explosion [74]. Replacing them with solid-state electrolytes not only makes it possible to solve the problems that have always existed with conventional liquid electrolytes, but also opens the door to inventing novel battery chemistries [75]. Solid-state electrolytes can be categorized into three main groups: inorganic solid electrolytes (ISEs) [76], solid-state polymer electrolytes (SPEs) [77], composite solid electrolytes (CSEs) and Plastic crystal electrolytes (PCEs).

### 4.1. Inorganic Solid Electrolyte

Inorganic solid electrolytes are amorphous solids primarily consisting of anions and cations, arranged in a way that enables unique ionic conductivity properties. ISEs mainly include oxides, sulfides, and halides [78]. The wettability between inorganic solid-state electrolytes and electrode materials is poor, and the interfacial impedance is usually high. Currently, inorganic solid-state electrolytes are less studied at LTs. The large radius of sulfur ions permits larger ion channels, which reduces the barrier during ion migration, consequently granting sulfide solid-state electrolytes excellent ionic conductivity, making them an important topic of research.

Peng et al. [79] synthesized chlorine-rich argyrodite electrolytes with high ionic conductivity (Li_5_._5_PS_4_._5_Cl_1_._5_ = 9.03 mS cm^−1^) via a simple solid-phase reaction method at RT. The introduction of chlorine, being a more electronegative element, alters the charge distribution within the crystal lattice, resulting in a more uniform electric field force experienced by lithium ions during their migration process. This uniform electric field favors the migration of lithium ions. Additionally, chlorine ions participate in the coordination, thereby creating a more spacious migration channel for Li^+^, which in turn reduces the migration energy barrier [80]. The NCM622//Li_5_._5_PS_4_._5_Cl_1_._5_//Li half-cell exhibited excellent cycling stability, with 82.4% capacity retention after 10,000 cycles at 10 C at RT. The capacity retention rate is 97.0% after 200 cycles at 0.2 C at a LT of −20 °C. This is due to the decomposition of the electrolyte to form polysulfides or oxides during the cycling process [81], which react with the electrode to form an SEI layer, thereby avoiding the continuation of the side reaction. This contributes to the improvement of the cycling stability and safety of the battery.

Li et al. [82] synthesized the LGPS-type sulfide solid-state electrolyte Li_9.54_[Si_1-δ_M_δ_]_1.74_P_1.44_S_11.1_Br_0.3_O_0.6_ (M = Ge, Sn; 0 ≤ δ ≤1) by partially replacing Si with the addition of Ge and Sn. LSiGePSBrO (M = Ge, δ = 0.4) showed an ultra-high ion conductivity of 32 mS cm^−1^ at RT and 9 mS cm^−1^ at −10 °C. The all-solid-state battery with a highly loaded cathode (800 μm thickness, mixed with LiNbO_3_-coated LiCoO_2_ (LNO-LCO)) in the LNO-LCO//LSiGePSBrO//Li half-cell exhibited a discharge capacity of 17.3 mAh cm^−2^ at −10 °C. Since the ions of different elements exhibit different arrangements and forces in the crystal, the ionic radii of Ge and Sn are slightly larger than that of Si, and their introduction leads to the expansion of the crystal volume and an increase in local disorder. By increasing the compositional complexity of the LGPS-type solid-state electrolyte by means of element substitution, ion migration can be facilitated while maintaining the structure of the ion-conducting framework [82].

### 4.2. Solid-State Polymer Electrolyte

Solid-state polymer electrolytes (SPEs) are a metal salt dissolved in a polar polymer matrix to form an ionic conductive phase. Compared to inorganic solid electrolytes, SPEs exhibit better mechanical flexibility. Consequently, SPEs are less prone to cracking at LT and better tolerant of low-temperature deformation, thereby improving battery safety [83,84]. However, the solid-state polymer electrolyte still faces challenges, such as low ionic conductivity, weak mechanical properties, and poor contact with the electrode interface. These problems are more apparent at LTs. To improve the ionic conductivity of SPEs, a common method is to add liquid electrolytes to the polymer to form a quasi-solid electrolyte (QSPE) [85,86].

**Figure 7:**
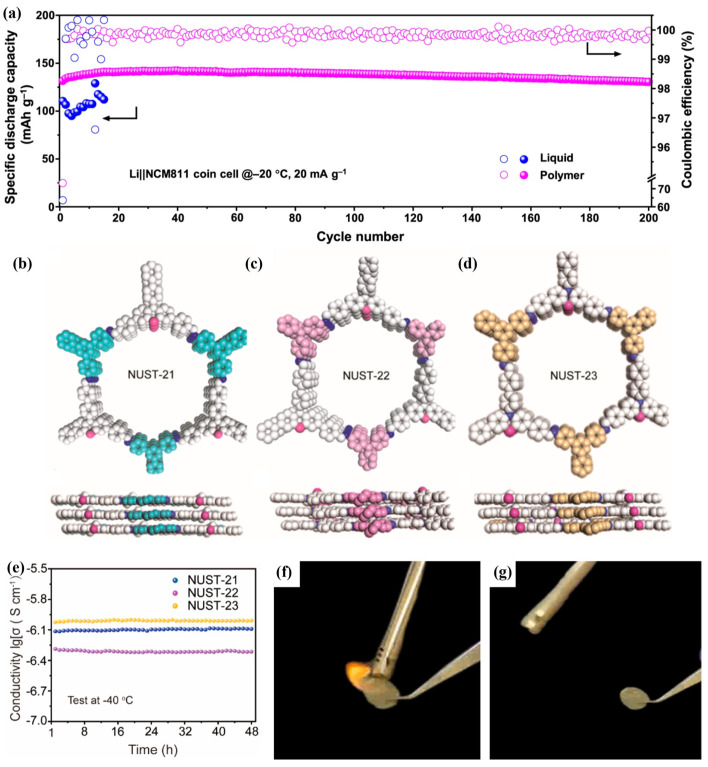
(**a**) Cycling performances of both electrolytes in Li//NCM811 half-cells at −20 °C and 20 mA g^−1^. (Reprinted with permission from [93]; copyright 2023, Nature Communications). (**b**–**d**) The top and side views of COFs: (**b**) NUST-21, (**c**)NUST-22, and (**d**) NUST-23. The experimental profiles are in black, calculated in blue, and the difference between the experimental and refined PXRD patterns is in green. (**e**) Long-period conductivity. (Reprinted with permission from [97]; copyright 2022, Chemistry of Materials). (**f**,**g**) Nonflammability test of the LiQSSE electrolyte through ignition. (Reprinted with permission from [100]; copyright 2024, Journal of Power Sources).

Considering that in-situ polymerization can provide good interfacial contact between QSPE and the electrodes [87,88], Ren et al. [88] added different lithium salts to 1,3-dioxolane (DOL) to obtain 2 M DOL/LiTFSI and 0.6 mM DOL/Al(OTf)_3_ (Aluminum trifluoromethanesulfonate) solutions, which were then mixed with the plasticizer FEC (2:8:1) to obtain precursor solutions at RT. The prepared precursor solution was left at RT for about 12 h to solidify to QSPE (Poly-DOL-10, FEC = 10 vol.%). When assembling the cell, the precursor solution was injected into the PP film, and the assembled cell was kept for about 12 h to complete the in-situ ring-opening polymerization process at RT. Poly-DOL-10 exhibited higher ionic conductivity in the range of −30~−60 °C compared to conventional liquid electrolytes. The ionic conductivity of Poly-DOL-10 at −60 °C is 2.4 × 10^−2^ mS cm^−1^. The decrease in ionic conductivity of liquid electrolytes at LT is due to the sudden increase in their viscosity [89]. In contrast, the incorporation of FEC in Poly-DOL-10 enhances the polymer chains motility during Li^+^ migration, leading to high ionic conductivity. The Li^+^ transference numbers of Poly-DOL-10 were 0.445, 0.547, and 0.579 at −60 °C, −20 °C, and 0 °C, respectively, which were greater than that of liquid electrolytes (1.0 M LiPF_6_ in EC/DEC (1:1 by volume) (0.073, 0.167, and 0.221, respectively). This is due to the high dielectric constant of FEC, which promotes the dissociation of Li^+^ [90,91,92]. Li et al. [93] dissolved 1 M LiDFOB as the lithium salt and initiator in a mixture of 1,3,5-trioxane monomer (TXE), 2,2,2-trifluoro-N,N-dimethylacetamide (FDMA), and FEC solvent (mass ratio of 5:3:1) to obtain the precursor solution. When assembling the battery, the precursor solution was injected into the Al_2_O_3_-coated PE separator and was kept at 80 °C for 2 h to achieve spontaneous in-situ polymerization of the QSPE (LiDF-FDMA-TXE). TXE has lower HOMO (highest occupied molecular orbital) and higher LUMO (lowest unoccupied molecular orbital) to improve oxidative and reductive stability [94]. At −20 °C, the LiDF-FDMA-TXE has a high ionic conductivity of 0.22 mS cm^−1^ and a high ion transference number of 0.8. After 200 cycles at −20 °C and 20 mA g^−1^, the Li//QSPE//NCM811 half-cell can maintain a high capacity of ~151 mAh g^−1^ (Figure 7a). Additionally, the LiDF-FDMA-TXE shows good low-temperature performance with the LiFePO_4_ cathode. The Li//LFP cell retains ~95 mAh g^−1^ over 350 cycles at −20 °C and 17 mA g^−1^. The Li//NCM811 pouch cell, featuring a single-side coated Li anode with a thickness of 50 μm and a single-side coated NCM811 cathode with a mass loading of 3 mg cm^−2^, maintains a capacity of about 148 mAh g^−1^ at −20 °C and 94 mAh g^−1^ at −30 °C over more than 10 cycles at a current density of 20 mA g^−1^.

Polymer electrolytes with covalent organic frameworks (COFs) have also been investigated to improve the electrochemical performance at LTs. COFs are formed by lightweight organic molecular building blocks connected by fully covalent bonds [95,96]. The porous structure of COFs provides more ion transport channels, which can alleviate the decrease in ionic conductivity of the electrolyte to some extent. Xuan et al. [97] prepared the electrolyte (Li^+^-PEG) by mixing polyethylene glycol (PEG) and LiTFSI (according to a [O]/[Li] molar ratio of 16:1). The COF powder (NUST-21) was obtained by mixing aldehyde monomers containing phenothiazine units (PT-CHO, 17.75 mg), 5′-(4-aminophenyl)-[1,1′:3′,1″-terphenyl]-4,4″-diamine (TAPB, 10.63 mg), o-Dichlorobenzene (o-DCB, 0.6 mL), 1,4 n-Butanol (1.4 mL), and 6M AcOH (0.2 mL). After removing oxygen and other impurity gases, the mixture was left under under nitrogen protection at 120 °C for 6 days. NUST-22/23 was obtained via the same method using different monomers (N1,N1-bis(4-aminophenyl)benzene-1,4-diamine (TAPA, 8.71 mg) and 4,4′,4″-(1,3,5-triazine-2,4,6-triyl)trianiline (TAPT, 10.6326 mg)). The novel macropores could allow for low-resistance transport of Li^+^ as well as absorb more electrolyte (Figure 7b–d). Li^+^-PEG@NUST-21/22/23 quasi-solid electrolytes were prepared by pressing COF powder and Li^+^-PEG (mass ratio = 1:1) together by mixing and grinding for 10 min in an argon-filled glove box. The ionic conductivity of Li^+^-PEG@NUST-21/22/23 at −40 °C were 7.55 × 10^−7^ S cm^−1^, 4.63 × 10^−7^ S cm^−1^, and 9.74 × 10^−7^ S cm^−1^, respectively. When stored at −40 °C for 48 h, the ionic conductivity of the three remained at 8.11 × 10^−7^ S cm^−1^, 4.27 × 10^−7^ S cm^−1^, and 9.79 × 10^−7^ S cm^−1^, respectively (Figure 7e), which is attributed to the flowable network formed by PEG chains and rigid COF structures confining Li^+^-PEG.

Chen et al. [98] obtained a conductive polymer electrolyte (SI10-05-70% PC; SI stands for Single-Ion Conductor) by mixing polyethersulfone (PES) and polyethersulfone (FPES) in a mass ratio of 10:5 and adding 70 wt.% PC into the copolymer. This electrolyte was copolymerized by the FPES ionophobic blocks and the ionophilic PES block with a lithium perfluorosulfonimide side chain. The flexibility and ionic conductivity were improved by adding 70 wt.% PC. By optimizing the mass ratio of PES: FPES to 10:5, the electrolyte was successfully made to exhibit high ionic conductivity over a wide range of temperatures (−30 °C to 90 °C), especially reaching 6 × 10^−4^ S cm^−1^ at 20 °C and >10^−4^ S cm^−1^ at −30 °C. In addition, the high fluorine element in FPES electronegativity makes the polymer less likely to decompose at high temperatures, and thus SI10-05-70%PC also exhibits nonflammability. They prepared lithium metal batteries by combining a SI10-05-70%PC electrolyte with a NCM811 cathode. The electrochemical results showed that the Li//SI10-05-70%PC//NCM811 battery was able to perform 500 cycles at a rate of 0.5 C at 0 °C and maintain a capacity of 109 mAh g^−1^.

Shi et al. [99] obtained a polymer electrolyte (PTFSI-10/5-PC, hydrophobic block molecular weight 5000 g mol^−1^, ionic block backbone molecular weight 10,000 g mol^−1^, PC with 70 wt.%) via bromination of the monomers that make up the copolymers and mixing them with LiTFSI, based on Chen et al. [98]. The ionic conductivity of PTFSI-10/5-PC reached 2.15 × 10^−4^ S cm^−1^ at 20 °C and 8.98 × 10^−5^ S cm^−1^ at 0 °C. The rate performance of the Li//PTFSI-10/5-PC//NMC622 cell decayed with an increasing rate when cycling at 0 °C, but a high capacity could still be obtained when recovering to a low C-rate (~80 mAh g^−1^ at 0.5 C). The Li//PTFSI-10/5-PC//NMC622 cell was able to maintain a stable cycling rate of 300 cycles at 0.5 C at 0 °C (~80% capacity retention, initial capacity of ~75 mAh g^−1^).

Das et al. [100] cast a mixed solution of acetone and poly (vinylidene fluoride-hexafluoropropylene) (PVDF-HFP) onto a non-woven (NW) sheet and subsequently dried it in a vacuum oven at 70 °C for 24 h to obtain a solid polymer mat (NW-SPM). This mat was then immersed in a liquid electrolyte (1M LiPF_6_ in MP/TMP/FEC) to form a nonwoven gel-based polymer electrolyte, referred to as LiQSSE. The ionic conductivity of LiQSSE was 3.9 × 10^−5^ S cm^−1^ at −20 °C. This excellent ionic conductivity at LT is due to the introduction of liquid electrolyte. MP, TMP, and FEC help to lower the freezing point of the electrolyte so that it remains liquid at LT, thus maintaining a high ionic transport capacity. Additionally, the LFP//LiQSSE//Li half-cells exhibited 93% capacity retention after 90 cycles at −10 °C with a rate of 0.5 C. The liquid electrolyte undergoes gelation in the three-dimensional network structure formed by the PVDF-HFP and the nonwoven fabric, forming a stable gel-based polymer electrolyte. This gelation process not only enhances the mechanical strength of the electrolyte, but also improves the long-term stability of the electrolyte by reducing solvent volatilization and leakage through solvent immobilization. In addition, the LFP//LiQSSE//graphite cell exhibits a distinct heat absorption peak at 180 °C, indicating electrolyte shrinkage. Small heat absorption peaks exhibited at around 165 °C and 140 °C indicate evaporation of the solvent used. In contrast, the NW used in LIQSSE has a high melting point (~171 °C), and the heat released by the LFP//LiQSSE//graphite full-cell is only 37 J g^−1^ (LFP//Celgard//graphite full-cell releases of 1.5 kJ g^−1^, commercial separator), which significantly enlarges the window of thermal stabilization. Ignition testing reveals the good non-flammability of the LiQSSE, as shown in Figure 7f,g.

In summary, the mobility of polymer chain segments decreases at LT, resulting in impeded ion transport. The introduction of organic solvents keeps some of the solvent molecules mobile at LT, thus maintaining the ion transport channels open. Preparation of electrolytes using an in-situ polymerization process improves the morphological structure of the polymer as well as the formation of ion channels. Introducing covalent organic frameworks with specific functionalities into polymer solid-state electrolytes can lead to the formation of more complex phase structures and improved ionic transport pathways, thereby increasing ionic conductivity. The use of high melting point nonwoven materials as the substrate for the solution casting method effectively enlarges the thermal stabilization window of the cell and improves the flame retardancy.

### 4.3. Composite Solid Electrolytes

Composite solid electrolytes (CSEs) are usually a composite of inorganic filler and polymer electrolyte, which combine the high ionic conductivity and mechanical strength of the inorganic filler with the good flexibility of the polymer substrate, thus making it easier to form a good interfacial contact with the electrode. Therefore, CSEs can potentially be an excellent alternative to lithium-ion battery electrolytes with good low-temperature performance and high safety.

**Figure 8:**
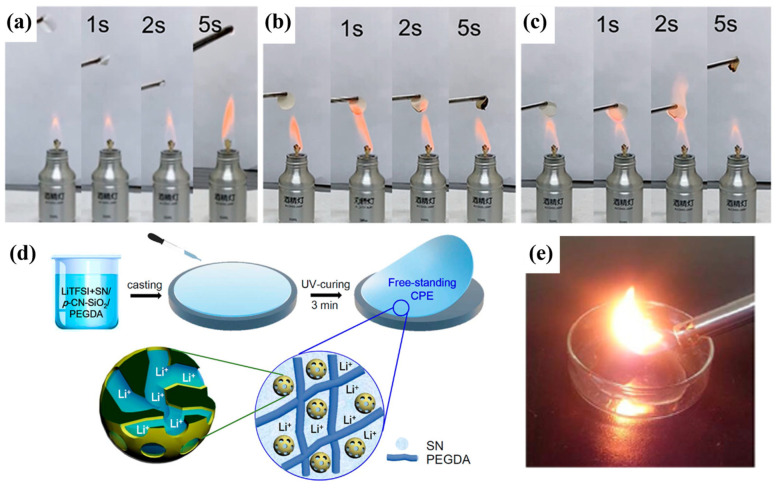
(**a**,**b**) The fire retardancy test of the (**a**) commercial Celgard separator, (**b**) pristine PPPL-10 membrane, and (**c**) PPPL-10 membrane after battery disassembly. (Reprinted with permission from [101]; copyright 2023, ACS Applied Materials & Interfaces). (**d**) Schematic illustration of composite polymer electrolytes (CPE) fabrication using p-CN-SiO_2_ nanoparticles and UV curing. (**e**) The test of the flame-retardant characteristics of the CPEs. (Reprinted with permission from [106]; copyright 2017, ACS Applied Materials & Interfaces).

Wang et al. [101] reported a CSE consisting of polydopamine (PDA)-coated Li_6.4_La_3_Zr_1.4_Ta_0.6_O_12_ (LLZTO) (denoted as PDA@LLZTO) microfiller, polyacrylonitrile (PAN), and poly(vinylidene fluoride-co-hexafluoropropylene) (PVDF-HFP), namely PPPL-10 (10% PDA@LLZTO powder). The CSE has good flame resistance compared to commercial separators (Figure 8a–c). This is because PAN has a high melting point of 317 °C, which can maintain structural stability at high temperatures and is not easily decomposed or melted. Therefore, PAN improves the thermal stability of electrolytes when introduced into CSEs. Compared to the LFP//Celgard//Li half-cell offering an initial capacity of 93.6 mAh g^−1^, the LFP//PPPL-10//Li half-cell provides a high initial capacity of 108.9 mAh g^−1^ at a rate of 0.2 C at 0 °C and has very high cycling stability, with 99.9% capacity retention after 100 cycles. During the decrease in test temperatures, the capacity of the LFP//PPPL-10//Li half-cell was 152.9 mAh g^−1^ (at RT), 119.7 mAh g^−1^ (at 10 °C), 108.6 mAh g^−1^ (at 0 °C), 76.7 mAh g^−1^ (at −10 °C), 44.8 mAh g^−1^ (at −20 °C), and 10.7 mAh g^−1^ (at −30 °C). During the gradual recovery of temperature to RT, the capacity is almost completely restored.

Yang et al. [102] designed a CES consisting of Li_1.4_Al_0.4_Ti_1.6_(PO_4_)_3_ nanowires (LNs), poly(vinylidene fluoride) (PVDF) and N,N-dimethylformamide (DMF), known as PVLN-15 (15 wt.% LNs). Multi-ion synergistic transport of DMF solvents and fillers in the CES contribute to the formation of a stable interface at the lithium metal anode and reduce the interfacial impedance. The PVLN-15 electrolyte has the highest RT ionic conductivity of 6.0 × 10^−4^ S cm^−1^, which is about two times higher than that of the pure PVDF electrolyte (2.9 × 10^−4^ S cm^−1^). This is because the DMF-coated LNs with PVDF polymers generate multiple Li^+^ transport channels, which reduce the Li^+^ transport resistance at the electrolyte/electrode interface. At a LT of −20 °C, the NCM811//PVLN-15//Li half-cell performed at a capacity of 168 mAh g^−1^ after 100 cycles at 1C. In contrast, the NCM811//PVDF//Li half-cell exhibits a capacity of merely 45 mAh g^−1^, ultimately resulting in a short circuit after 47 cycles. This is because the LNs anchor the DMF in the electrolyte, significantly increasing the upper limit of the electrochemical window of the PVLN-15 electrolyte from 4.3 V to 4.5 V and inhibiting the decomposition of the DMF at the PVLN-15 electrolyte/lithium metal interface.

Later, the research team of Bresser & Passerini reported the use of Nano-ZnO-PEO (poly(ethyleneoxide)) hybrid polymer electrolytes and observed that the ionic conductivity increased with an increasing ZnO content [103]. This work has drawn much attention to the role played by nanoparticles in polymer electrolytes. Claudio Gerbaldi’s group [104] crosslinked PEO as a polymer with HPyr by ultraviolet (UV) curing to obtain the PEO_HPyr polymer electrolyte (HPyr is a mixture of PIL 1-butylpyrrolidinium bis(trifluoromethanesulfonyl)imide (Pyr_H4_TFSI) and LiTFSI). The ionic conductivity of pure Pyr_H4_TFS, HPyr, and PEO_HPyr was also tested in the range of −20 °C to 80 °C. The ionic conductivities of PEO_HPyr were all found to be lower than those of pure Pyr_H4_TFS or HPyr. Because PEO is a semi-crystalline polymer, the crystallization of PEO leads to a reduction in the channels for ion migration in PEO_HPyr composites. However, only PEO_HPyr was able to maintain a relatively high ionic conductivity at lower temperatures (−20 °C to 0 °C). This demonstrates that the UV cross-linking structure helps to prevent structural collapse of the electrolyte, thereby maintaining long-term ionic transport capacity.

The in-situ polymerization method, which has been investigated in SPEs, is able to optimize the electrolyte structure, as well as improve ionic conductivity and interfacial stability. Lee et al. [105] prepared CSEs (P(TMC_80_CL_20_)-LiTFSI_0.28_-ZrO_2_ (4 wt.%)) by compositing LiTFSI, copolymers P(CL_80_TMC_20_) (80 mol % ϵ-caprolcatone (CL) and 20 mol % trimethylene carbonate (TMC)) and zirconia (ZrO_2_) nanoparticles via the solvent-gel method. Different samples were prepared using in-situ and ex-situ methods. From the results of ionic conductivity of P(TMC_80_CL_20_)-LiTFSI_0.28_-ZrO_2_ (4 wt.%) at 30 °C, it was found that the ionic conductivity of the ex-situ method (5.6 × 10^−6^ S cm^−1^) was lower than that of the in-situ method (5.25 × 10^−5^ S cm^−1^). This further explains the better dispersion of nanoparticles in the in-situ polymerization method. The in-situ UV-curing process may be more advantageous in terms of low-temperature performance enhancement in CSEs. Kwon’s team [106] prepared precursor solutions by mixing function-specific nanoparticles (nitrile-functionalized silica nanoparticles (CN-SiO_2_) or porous nitrile-functionalized silica nanoparticles (p-CN-SiO_2_)) and polymers (PEGDA). The precursor solution was then mixed with an electrolyte (3.4 M LiTFSI + succinonitrile (SN)), followed by the addition of two photoinitiators (2-hydroxy-2-methyl-1-phenyl-1-propane (HMPP) and Lucirin TPO (BASF)). After being ball-milled for 1 h, composite polymer electrolytes (CPEs) were obtained by casting and irradiation under a UV lamp for 3 min. The schematic of CPE preparation using the UV curing method is shown in Figure 8d. The in-situ UV-curing process causes cross-linking reactions of polymer monomers through photoinitiators, thus achieving rapid curing of CSEs under mild conditions, which avoids the high temperature conditions required by the traditional heat-curing method and facilitating the maintenance of the stability and performance of each component in the CPEs. As shown in Figure 8e, the CPE exhibits good non-flammability. The abundant mesoporous structure of CN-SiO_2_ nanoparticles provides additional transport channels for Li^+^, which makes the transport paths of Li^+^ in the electrolytes more diverse. The specific surface area of CN-SiO_2_ nanoparticles is increased by the mesoporous structure. The increase in specific surface area means that more Li^+^ can interact with the surface of the electrolyte, which promotes Li^+^ conduction. The strong coordination between the nitrile group (-CN) and Li^+^ also contributes to the formation of a stable solvation structure [107]. This stable structure can potentially reduce the impediments to ionic motion at LT, thereby improving the ionic conductivity of the electrolyte. The electrolyte has an ionic conductivity of up to 2 × 10^−3^ S cm^−1^ at RT and maintains an ionic conductivity of >10^−4^ S cm^−1^ at subzero temperatures. The LTO//CSE//Li half-cell exhibited a stable discharge capacity of 151 mAh g^−1^ at temperatures below −10 °C, which corresponds to 92% of the capacity at RT (164 mAh g^−1^).

Composite solid electrolytes (CSEs) combine the high ionic conductivity and mechanical strength of inorganic fillers with the flexibility of polymers, ensuring better resistance to external shocks and reducing interfacial resistance and side reactions while maintaining high capacity even at subzero temperatures. In addition, the use of specially structured inorganic fillers and novel processing techniques allows for fast curing under mild conditions, thus simplifying processing steps and improving low-temperature performance. In summary, CSEs are promising alternatives to conventional electrolytes for LIBs, with excellent low-temperature performance and enhanced safety.

### 4.4. Plastic Crystal Electrolytes

The Plastic Crystal Electrolyte (PCE) is a special classification of solid electrolytes. Within a certain temperature range, the molecular or ionic arrangement of PCEs maintains an ordered structure (like crystals) and exhibits a certain degree of plasticity [108]. Plastic crystals have higher diffusivity and plasticity, which facilitates the transfer of ions in them, thus improving the overall performance of solid-state electrolytes.

In the process of deeply exploring the field of SSEs with the aim of achieving breakthroughs in high-performance battery technology, scientists are actively seeking innovative materials and engaging in advanced structural design exploration. Among these endeavors, PCEs—as a novel class of electrolyte materials—have emerged as one of the key focuses for researchers. Wang et al. [109] confined organic plastic crystals within COFs to prepare CSEs for ultra-fast ionic conduction utilizing the entropy-driven effects. TPB-DMTP-COFs were synthesized under solvothermal conditions using 1,3,5-Tris(4-aminophenyl)benzene (TPB) and 2,5-dimethylterephthalaldehyde (DMTP). Vacuum treatment was utilized to permeate Tetrabutylphosphonium bis(trifluoromethyl sulfonyl)imide (PBu_4_TFSI) into the pores of the TPB-DMTP-COFs, and then mixed with LiTFSI, resulting in a composite named LiPBu_4_TFSI@TPB-DMTP-COF. This electrolyte (LiPBu_4_TFSI@TPB-DMTP-COF) exhibits a high Li^+^ conductivity of 0.048 S cm^−1^ at 30 °C and 0.021 S cm^−1^ at −30 °C. This is attributed to the ability of plastic crystals to undergo a phase transition from the solid phase to the plastic-crystalline phase in response to temperature changes. In the plastic-crystalline phase, the molecular/ionic motion of plastic crystals is enhanced, and the number of vacancies in the lattice that accompany their motion increases, both of which are favorable for Li^+^ transference. Therefore, at LT, the plastic crystals can maintain or partially maintain the plastic-crystalline phase, which helps to improve the LT performance of the battery.

**Figure 9:**
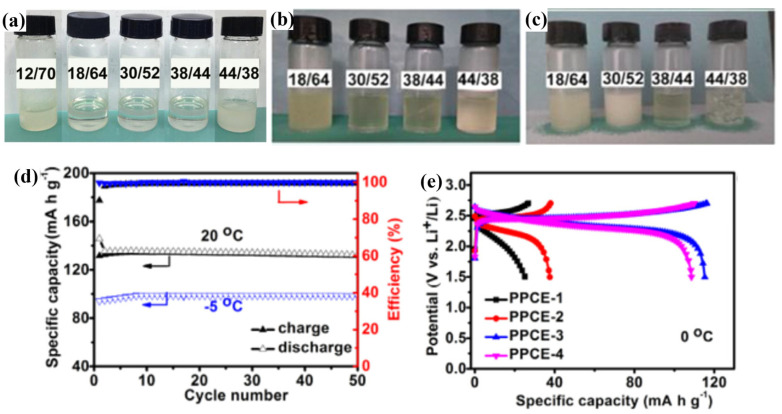
(**a**) Photographs of the five PCE mixtures at 20 °C, which had the same LiTFSI/SN mass ratios as those of the five PPCEs. (**b**,**c**) Photographs of the four PCE mixtures maintained at different temperatures for 0.5 h: (**b**) 0 °C and (**c**) −40 °C. (**d**) Cyclic stabilities of the LCO/LTO cell with PPCE-3 at the current density of 75 mA g^−1^ at 20 °C and −5 °C. (**e**) Characteristic charge/discharge voltage profiles of the LCO/LTO cells with the five PPCEs at a current density of 75 mA g^−1^ at 0 °C. (Reprinted with permission from [110]; copyright 2020, Journal of Energy Chemistry).

SN is a typical plastic crystal commonly used to prepare LT PCEs. Zhou et al. [110] prepared polymer plastic crystal electrolytes (PPCEs) by mixing LiTFSI, SN and PVDF-HFP. Five plastic crystal electrolytes with different ratios, labeled PPCE-1 to PPCE-5, were prepared, in which the content of PVDF-HFP was fixed at 18 wt.% and the total content of LiTFSI and SN was 82 wt.% (the proportions of LiTFSI and SN varied, from 12:70, 18:64, 30:52, 38:44, and 44:38 in weight respectively). Among them, PPCE-1 showed a waxy solid state, whereas PPCE-2 through PPCE-4 were in transparent solution form, and PPCE-5 was in a translucent suspension (Figure 9a). PPCE-3 exhibited the highest ionic conductivity (6 × 10^−4^ S cm^−1^) at 0 °C and −20 °C, as it still maintains a partially liquid state (Figure 9b,c). At 20 °C, the LCO//PPCE-3//LTO full-cell was able to exhibit the highest specific capacity (135 mAh g^−1^) at a current density of 72 mA g^−1^ (Figure 9d). At 0 °C, the specific capacity of the LCO//PPCE-3//LTO full-cell remained at a high level (115 mAh g^−1^) (Figure 9e). In addition, the specific capacities of LCO//PPCE-3//LTO full-cells, which were subjected to multiplication performance tests (15, 30, 75, 150 and 300 mA g^−1^) at −5 °C, were 128 mAh g^−1^, 120 mAh g^−1^, 98 mAh g^−1^, 73 mAh g^−1^, and 46 mAh g^−1^, respectively.

With the continuous development of material science and battery technology, plastic crystal electrolytes are expected to further improve the performance and stability of these plastic crystal electrolytes by optimizing the material composition, preparation process and structural design, to promote their application and development in the field of high energy density and high-safety batteries.

In summary, ISEs, SPEs, CSEs, and PCEs have their own advantages in LT applications. ISEs have high ionic conductivity but it is necessary to consider the optimization of the interfacial reaction to ensure their safety; SPEs have good flexibility and interfacial stability, which can enhance the structural safety of the battery, but the problem of low conductivity at LT still needs to be continuously researched; CSEs combine the advantages of both and have made significant progress in ionic conductivity and cycling stability. By optimizing the material composition and structural design, such as with PCEs, this approach enhances the compatibility between electrolyte and electrode, and further improves the overall safety of the battery. All this research has effectively mitigated the risk of solidification and internal short-circuiting of traditional liquid electrolytes at LT, and at the same time laid a foundation for the wide application of low-temperature LIBs.

## 5. Ionic Liquid-Based Electrolytes

Ionic liquids (ILs) consist of organic cations and inorganic/organic anions, which are ionic compounds that are liquid at or near RT. ILs have also been considered as alternatives to conventional organic electrolytes due to their high electrochemical stability, high thermal stability, negligible vapor pressure, and non-flammability [111,112]. ILs can be directly used as solvents in electrolytes, but ILs usually have high viscosity, low ionic conductivity, and poor compatibility with graphite anodes. Therefore, the application of pure ILs as LIB electrolytes is limited [113,114,115]. Many strategies have been adopted to address these issues, such as the use of blended ionic liquids, the addition of co-solvents with dilution functions based on high lithium salt concentrations, and the direct addition of co-solvents [116,117].

Moreno et al. [118] dissolved LiTFSI salts in a mixture of two ionic liquids with the same cation (N-methyl-N-propyl pyrrolidinium cation (PYR13^+^)) but different anions (bis (trifluoromethane-sulfonyl)imide (TFSI^−^) and bis(fluorosulfonyl)imide (FSI^−^)) to obtain a ternary electrolyte (PYR_13_TFSI-PYR_13_FSI). After adjusting the ratio of FSI/TFSI (molar ratio of 3:2), the ionic conductivity of the ILs was about 10^−3^ S cm^−1^ at −20 °C. This can be attributed to the distinct steric hindrance posed by the TFSI and FSI anions, effectively impeding the crystallization of the ionic liquid mixtures. With the increase in LiTFSI content, the ionic conductivity of the electrolyte did not change significantly at 20 °C but decreased gradually at −20 °C; this is due to the enhancement of ion interaction at LT, resulting in increased viscosity and increased resistance. In a word, by blending different ionic liquids and adjusting their ratios, it is expected to obtain ILs with good low-temperature performance.

**Figure 10:**
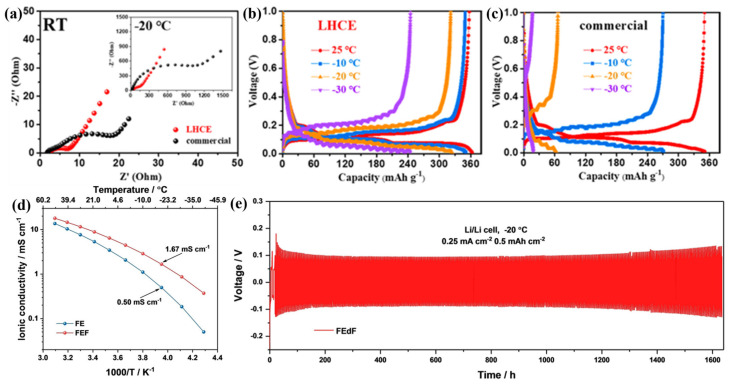
(**a**) EIS of Gr//Li batteries after 20 cycles at RT; the inset is the EIS at −20 °C. Charge/discharge plots of Gr//Li batteries when cycled at 25, −10, −20, and −30 °C in (**b**) LHCEs and (**c**) commercial electrolytes. (Reprinted with permission from [119]; copyright 2022, ACS Sustainable Chemistry & Engineering). (**d**) Ionic conductivity of FE and FEdF at various temperatures. (**e**) Voltage profile of a Li/FEdF/Li battery upon a long-term, galvanostatic plating/stripping cycling test. (Reprinted with permission from [126]; copyright 2022, Advanced Energy Materials).

To solve the problem of poor compatibility between ILs and graphite, Wang et al. [119] dissolved 0.9 M LiFSI in an ionic solution ([PP^+^_13_][FSI^−^]) and then added a non-polar solvent (HFE) to obtain localized, highly concentrated electrolytes (LHCEs). In LHCEs, the anions (FSI^−^) of the solvent molecules are almost always coordinated with the Li^+^ of the highly concentrated lithium salt to form aggregates. HFE does not coordinate with Li^+^, and its incorporation reduces the overall salt concentration of the electrolyte while preserving the local coordination environment of the highly concentrated salt-solvent clusters. This IL-based LHCE maintains the excellent performance of the highly concentrated electrolyte while reducing its viscosity and cost and remaining liquid at −30 °C. Wang et al. [119] tested the graphite//LHCE//Li half-cell for 300 cycles at a current density of 3 C at RT. It was found that the discharge capacity was about 2.5 times higher than that of one using a commercial carbonate electrolyte (190 mAh g^−1^/75 mAh g^−1^). The batteries using LHCEs have better cycling stability, probably due to the unique solvation structure of LHCE, which can produce a thin, uniform, and strong inorganic SEI layer on the graphite anode surface, effectively inhibiting cation co-intercalation in the graphite anode. The lower interface impedance of the graphite//LHCE//Li half-cell is lower compared to that of the cell based on a commercial carbonate electrolyte (1 M LiPF_6_ in EC/DEC (1:1 vol.%)) at both RT and −20 °C (Figure 10a). As shown in Figure 10b,c, compared to the half-cell based on the commercial carbonate electrolyte, the LHCE-based half-cell exhibited excellent LT reversible capacities of 352, 325, and 247 mAh g^−1^ at −10 °C, −20 °C and −30 °C, respectively.

Due to the inherently high viscosity of ILEs, Li^+^ transport becomes worse at lower temperatures [120]. In order to solve the above problems, based on the concept of concentrated electrolytes [121,122,123,124], researchers have formed locally concentrated ionic liquid electrolytes (LCILEs) by diluting ionic liquid electrolytes with low-viscosity and non-solvating co-solvents [121,125,126,127]. Liu et al. [128] synthesized an LCILE with LiFSI, 1-ethyl-3-methylimidazolium bis(fluorosulfonyl)imide (EmimFSI), and 1,2-difluorobenzene (dFBn) (in molar ratio of 1:2:2), which was termed FEdF. As a low melting point co-solvent, dFBn, on the one hand, reduced the solution viscosity and facilitated Li^+^ transport (the ionic conductivity increased from 5.28 × 10^−3^ S cm^−1^ to 8.84 × 10^−3^ S cm^−1^ at 20 °C). On the other hand, the poor compatibility of the electrode/electrolyte interface between the Li metal anode and the NMC811 cathode was improved. In the long-cycle test at RT, the capacity of Li//FE//NMC811 cells rapidly decayed from 168 mAh g^−1^ to 49 mAh g^−1^ after 250 cycles at 1 C. In contrast, the Li//FEdF//NMC811 battery provided 179 mAh g^−1^ after 500 cycles at 1 C with 93% capacity retention. This excellent cycling stability demonstrates the excellent compatibility of FEdF with the Ni-rich NMC811.

In a follow-up study, they [126] evaluated the effect of non-solvated co-solvents on the performance of Li//FE//NMC811 cells at LT. Since solid electrolytes are one of the problems leading to poor electrochemical performance at LT, it was found that no flash was detected for FE in flash point tests over the temperature range of 25–300 °C. The addition of dFBn with a flash point of 1 °C leads to the occurrence of a flash at 114 °C for FEdF, and therefore FEdF can be classified as having low flammability. The ionic conductivity of FE and FEdF decreased with decreasing temperature in the range of −40 °C to 50 °C. The ionic conductivities of FE and FEdF are 5 × 10^−4^ S cm^−1^ and 1.67 × 10^−3^ S cm^−1^ at −20 °C (Figure 10d), respectively, and these excellent ionic conductivities may be attributed to the fact that FE and FEdF remained liquid at −80 °C. In addition, the Li//FEdE//Li cell was tested at 0.25 C and −20 °C. As shown in Figure 10e, a decrease in the overvoltage was observed in the initial 25 cycles, after which the stable overvoltage was maintained for more than 1000 h. The results demonstrate that the lithium intercalation/de-intercalation in LCILE is unaffected at LT.

In conclusion, the performance of ionic ILs at LT is affected by various factors, such as viscosity and solvation structure. The liquid phase temperature range and conductivity can be expanded by mixing different ionic liquids. In addition, diluting part of the solvent in the high concentration electrolyte to form a LHCE can combine the advantages of high ionic conductivity, high nonflammability, and good fluidity at LT. Therefore, ILs are also an important topic for expanding the application of LIBs in the future.

## 6. Liquid Inorganic Electrolytes

Liquid inorganic electrolytes are electrolyte systems consisting of inorganic compounds capable of ionizing into ions in the liquid state as the solute, with water or non-aqueous as the inorganic solvent. Inorganic compounds can dissociate into free solvent molecules in solution and thus have good electrical conductivity. Inorganic liquid electrolytes can be categorized according to their solvents, which are mainly divided into two categories: aqueous electrolytes and non-aqueous electrolytes.

### 6.1. Aqueous Electrolytes

Aqueous electrolytes are electrolytes made of inorganic acid and alkali salts as solutes and water as the solvent, which are uniformly mixed [129]. Due to the good electrical conductivity of water molecules, aqueous electrolytes usually have a high conductivity; electrochemical process can quickly transfer the charge, thus improving the performance of the battery. Moreover, compared with organic electrolytes, aqueous electrolytes have higher safety and are not flammable or explosive. However, water as a solvent is prone to freezing at low temperatures, resulting in poor fluidity of the electrolyte, which affects the low-temperature performance of the battery.

To enhance the LT performance of aqueous LIBs, water-in-salt electrolytes (WISEs) have been investigated. Becker et al. [130] proposed mixing a novel lithium salt with asymmetric (pentafluoroethanesulfonyl)(trifluoromethanesulfonyl)imide (PTFSI) anion with lithium trifluoromethanesulfonate (LiOTf). The asymmetric PTFSI anion is a hybrid between the symmetric TFSI and bis(pentafluoroethanesulfonyl)imide (BETI) anions. The addition of LiOTf resulted in an increase in anionic species; the mixing entropy increases, and the electrolyte liquid phase temperature decreases to −14 °C (the mole ratio of LiPTFSI to LiOTf is 15:5, abbreviated 15 m:5 m). They assembled LiMn_2_O_4_//LiPTFSI:LiOTf (15m:5m)//Li half-cells for charge/discharge tests (100 cycles) at 25 °C, 0 °C, and −10 °C. The LiMn_2_O_4_//21m LiPTFSI//Li half-cells were compared (21 m refers to the addition of 21 mol kg^−1^ of LiPTFSI). Both half-cells showed an initial capacity of 105–110 mAh g^−1^ when cycled at a rate of 1 C at 25 °C, with coulombic efficiencies ranging from 99.0 to 99.6% after 100 cycles. At 0 °C, the LiMn_2_O_4_//LiPTFSI:LiOTf(15 m:5 m)//Li half-cells maintained their high capacity (110 mAh g^−1^), while the capacity of the LiMn_2_O_4_//21m LiPTFSI//Li half-cells decreased (95 mAh g^−1^). In addition, the ohmic resistance of the LiMn_2_O_4_//21m LiPTFSI//Li half-cells more than doubled during cycling at 0 °C (100 Ω to 250 Ω), indicating that the electrolyte partially solidified. During cycling at −10 °C, only the LiMn_2_O_4_//LiPTFSI:LiOTf(15 m:5 m)//Li half-cells maintained a high capacity of >100 mAh g^−1^, while the LiMn_2_O_4_//21m LiPTFSI//Li half-cells had a significantly lower capacity (~70 mAh g^−1^). Like the 0 °C case, the resistance of the latter half-cells also increased significantly during cycling at −10 °C. In contrast, the resistance of the LiMn_2_O_4_//LiPTFSI:LiOTf(15m:5m)//Li half-cells decreased slightly during cycling at all test temperatures.

Traditional aqueous electrolytes and electrode materials are prone to side reactions, such as dissolution and corrosion, which limit the electrochemical stabilization window of LIBs. To address this limitation, Chen et al. [131] utilized a mixture of Water/Acetonitrile (W/AN) as the solvent for the electrolyte and a high concentration of LiTFSI as the solute to prepare an “AN/Water-in-Salt” electrolyte (AN-WISE). AN-WISE exhibits a low solidification point (−48 °C), a high oxidative stability (>5 V vs. Li^+^/Li), and high miscibility with water, enabling it to remain liquid at LT and possess high conductivity. The inclusion of AN co-solvents enhances the interactions between H_2_O molecules and Li^+^, thereby reducing the content of free H_2_O molecules and achieving an electrochemical stability window of 4.5 V. In the temperature range of −20 °C to 60 °C, the ionic conductivity of BSiS-A_0.25_ (17.5 M LiTFSI in mixture of water:AN (with a molar ratio 3:1)) was superior to that of BSiS-D_0.28_ (13.3 M LiTFSI in a mixture of water:DMC (with molar ratio 2.6:1)), particularly at LT. Specifically, at −20 °C, BSiS-A_0.5_ remained liquid and provided ionic conductivities of 1.34 mS cm^−1^ and 0.63 mS cm^−1^ at 0 °C and −20 °C, respectively. The LiMn_2_O_4_//BSiS-A_0_._5_//LTO full-cell maintained a discharge capacity of 110 mAh g^−1^ after 120 cycles at a current density of 1 C at 0 °C, with a capacity retention rate of 95%. In contrast, under the same test conditions, the battery performance of the LiMn_2_O_4_//BSiS-D_0.28_//LTO full-cell plummeted to nearly 0 mAh g^−1^.

Aqueous electrolytes do not contain organic solvents; they are highly non-flammable. While retaining the safety advantages of aqueous electrolytes, WISEs also enhance the overall battery performance by broadening the electrochemical window through an increase in the lithium salt concentration. However, the performance of WISEs is significantly influenced by the diverse nature of the anions used, and not all electrode materials are compatible for use in aqueous electrolytes. At LT, WISEs are prone to solute precipitation due to reduced solubility, which can decrease the battery’s cycling stability. The incorporation of organic solvents to reduce the viscosity of the electrolyte can potentially improve the battery’s LT performance, but this undoubtedly adds to the production complexity and cost.

### 6.2. Non-Aqueous Electrolytes

Sulfur dioxide-based inorganic electrolytes (IEs) are electrolytes featuring a high concentration of Li^+^, with a composition like ionic liquids [132,133]. It is characterized by high ionic conductivity, high Li^+^ transference number, non-flammability, and LT stability [134]. When applied as an electrolyte in LIBs, it exhibits good compatibility with the cathode/anode interface [135,136,137].

Dinger et al. [138] prepared an inorganic electrolyte from anhydrous lithium chloride (AlCl_3_), lithium chloride (LiCl), and SO_2_ gas in the last century. The conductivity of the inorganic electrolyte was 2.2 × 10^−3^ S·cm^−1^ at −25 °CC. Mews et al. [139] summarized the solvation reactions of alkali metal chlorides and anhydrous AlCl_3_ in a SO_2_ environment as Equation (1):

(1) $$MCl+{AlCl}_{3}+{nSO}_{2}\to M^{+}{\left[{AlCl}_{4}\right]}^{-}\cdot {nSO}_{2}$$

The inorganic electrolyte prepared by Stassen et al. [140] used the same approach. Nailing, heating (up to 160 °C/200 °C), and short-circuiting experiments were performed on 3 Ah and 50 Ah LiCoO_2_//Li cells assembled with inorganic electrolytes. No serious events such as fire were observed in any case, indicating the high safety of the inorganic electrolyte.

**Figure 11:**
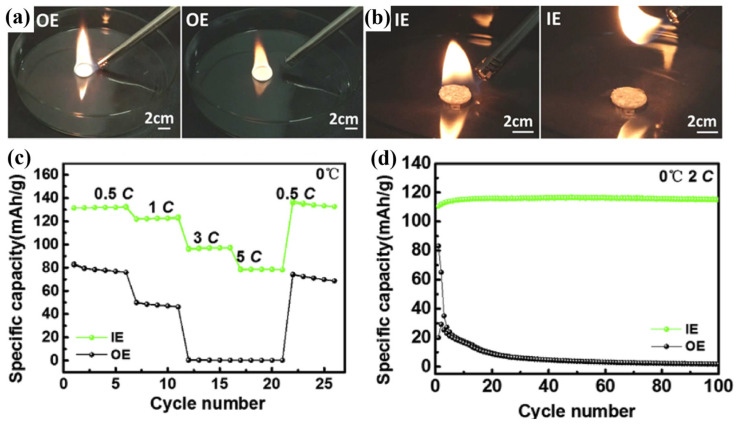
(**a**) Digital photos for ignition tests of different electrolyte-socked separators to be contacted with an open flame: (**a**) OE and (**b**) IE. Electrochemical performance of the LFP//Li half-cell at RT with different electrolytes. (**c**) Rate capabilities at C rates of 0.5 C, 1 C, 3 C, 5 C and 0.5 C. (**d**) Cycle performance at the rate of 2 C at 0 °C. (Reprinted with permission from [142]; copyright 2018, Electrochemical Acta).

To further improve the conductivity of inorganic electrolytes, Hartl et al. [141] prepared a LiAlCl_4_·1.6SO_2_ electrolyte by controlling the volume of SO_2_ gas. The Li^+^ transference number of this electrolyte was tested to be about 0.6. The LiAlCl_4_·3SO_2_ synthesized by Gao et al. [142] has an ionic conductivity of 2.377 × 10^−2^ S cm^−1^ at RT, and a Li^+^ transference number of 0.47. Compared to organic electrolytes (1.0 M LiPF_6_ in PC/EC/DEC (1:1:1 vol.%)), the LiAlCl_4_·3SO_2_ is nonflammable, as can be seen from the ignition test shown in Figure 11a,b. The assembled LFP//LiAlCl_4_·3SO_2_//Li half-cells were still able to discharge a capacity of about 80 mAh g^−1^ at RT at a high current density of 10 C. The battery capacity of the LFP//LiAlCl_4_·3SO_2_//Li after 100 cycles at 0.5 C at RT was 113 mAh g^−1^ (capacity retention 93.7%). The rate capability shown in Figure 9c shows that and the assembled LFP//LiAlCl_4_·3SO_2_//Li half-cells were tested at LT at 0 °C. The discharge capacities at 0.5 C, 1 C, 3 C, and 5 C were 131, 121, 96, and 78 mAh g^−1^, respectively, whereas the discharge capacities of the LFP//OE//Li cells at 0.5 C and 1 C were 83 and 50 mAh g^−1^, and the reversible capacity at 3 C and 5 C was almost 0. The LFP//LiAlCl_4_·3SO_2_//Li half-cells exhibited stable cycling performance at 2 C at 0 °C, with an increase in the discharge capacity from 110.6 mAh g^−1^ to 115 mAh g^−1^ after 100 cycles (Figure 11d). This slight increase could be attributed to the electrolyte and the active materials’ activation after charge/discharge cycles. In contrast, under the same test conditions, the discharge capacity of the LFP//OE//Li half-cells decreased dramatically, to 9 mAh g^−1^ at 20 cycles.

Subsequently, Gao et al. [143] continued to analyze the performance of LiAlCl_4_·3SO_2_ at LT. The ionic conductivity of the LiAlCl_4_·3SO_2_ was tested at different temperatures, and the ionic conductivity was 27.51 × 10^−3^ S cm^−1^, 11.04 × 10^−3^ S cm^−1^, and 6.54 × 10^−3^ S cm^−1^ at 0 °C, −10 °C, and −20 °C, respectively. The Coulombic efficiency of Li//LiAlCl_4_·3SO_2_//Cu was higher than that of Li//OE//Cu for any temperature condition tested at 0.5 mA cm^−2^. The Coulombic efficiency of LiAlCl_4_·3SO_2_ at −20 °C was about 95%, and the lower Coulombic efficiency of OEs is mainly due to the severe Li dendrite growth at LT. In contrast, LiAlCl_4_·3SO_2_ still deposits a uniform and dense SEI layer on the Cu foil after cycling at 0 °C.

Despite the superior performance of IEs, one of their feedstocks is highly reactive anhydrous AlCl_3_, which makes them susceptible to reactions with water vapor, oxygen, and so on. In a humid environment, anhydrous AlCl_3_ will rapidly absorb moisture in the air and form hydrates, thus changing its original chemical properties and physical state, leading to a decline in electrolyte performance or failure. LiCl also has a certain degree of hygroscopicity, and in a humid environment, it is easy to absorb moisture in the air, leading to an increase in the moisture content of the electrolyte, which in turn affects its conductivity and stability. This makes it necessary to strictly control the ambient humidity during both the preparation and storage of IEs to avoid performance degradation or failure. The SO_2_ gas itself is a toxic gas with an irritating odor, increasing the safety risk during production and use. Therefore, there is relatively little research on this electrolyte.

In summary, the inherent nonflammability of the inorganic liquid electrolyte itself makes it a strong guarantee for improving the safety of LIB applications. Since there is no organic solvent, the inorganic electrolyte is stable, nonvolatile, and has low viscosity, resulting in a wide range of low-temperature applications. Aqueous electrolytes increase the liquid range by increasing the lithium salt concentration and anionic species. The ionic conductivity of SO_2_-based electrolytes can be changed by adjusting the volume of SO_2_ gas passed through. With the deepening of research and the continuous development of technology, inorganic liquid electrolytes are expected to be applied in more fields, showing especially great potential in high energy density and long-life batteries.

## 7. Conclusions and Perspectives

**Figure 12:**
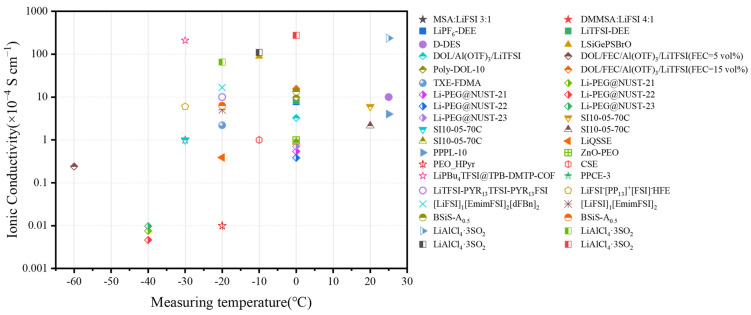
The ionic conductivity of different electrolytes at different temperatures [66,67,68,82,88,93,97,98,99,100,101,103,104,105,109,110,118,119,126,128,131,142,143].

This review describes the types and development of electrolytes for the application of high-safety LIBs at LT. The challenges of high safety LIBs at LT have been solved in many ways, but not completely, and still face many problems to be solved. Figure 12 summarizes the ionic conductivity of different electrolytes at different temperatures. The performance of LIBs with different electrolytes is summarized in Table 1.

In recent years, researchers have proposed many solutions to address the low-temperature performance of LIBs, but these solutions are not yet perfect.

(1) For traditional organic liquid electrolytes: Low melting point co-solvents can lower the melting point of the electrolyte, maintain fluidity at LT, accelerate ion migration, and improve LT conductivity. Some additives can form SEI films, lowering interfacial resistance and improving battery performance. A high concentration of single/mixed lithium salts reduces side reactions between the electrolyte and the electrodes, resulting in a longer cycle life of the battery. The convergence of the advantages of different lithium salts can improve the LT performance of the battery.
(2) For deep eutectic solvents-based electrolytes: They are mixtures that form eutectic structures through strong interactions and can keep the overall melting point of the solvent blend lower than that of its individual component solvents. This characteristic reduces the risk of electrolyte volatilization. Eutectic solvents typically exhibit an amorphous or locally ordered structure, enabling solvent molecules to easily rearrange and flow rather than forming a stable crystal structure at low temperatures, thus remaining liquid. However, the issue of increased battery interfacial resistance arising from the formation of a specific solvation structure between solvent molecules and lithium ions within the electrolyte remains a challenge to be addressed.
(3) For solid-state electrolytes: Solid-state electrolytes have the characteristics of non-flammability, high temperature resistance, and non-corrosion, which fundamentally eliminate the safety hazards caused by electrolyte leakage and electrode short-circuiting in traditional liquid electrolytes. This allows solid-state batteries to maintain a high level of safety even under extreme conditions. Methods such as copolymerization and the addition of plasticizers can improve the ionic conductivity of polymers by reducing their crystallinity, increasing the proportion of amorphous regions, and increasing the concentration of carrier ions. Plastic crystals with ordered crystal structures are chosen to make the electrolyte more malleable and promote efficient ion migration. A low-cost and efficient solution to the poor solid-solid contact of SSEs is still a problem that needs to be explored further.
(4) For ionic liquid electrolytes: Ionic liquid electrolytes usually have a low melting point and high ionic conductivity and are non-flammable, non-explosive, and have low volatility. However, the choice of raw materials for ionic liquid electrolytes affects their viscosity, and the compatibility between different ionic liquids and electrode materials needs to be constantly considered. Currently, ionic liquid electrolytes can show excellent performance under laboratory conditions, but their commercialization faces challenges such as process complexity and high production costs.
(5) For inorganic liquid electrolytes: Aqueous electrolytes, whose main components are water and electrolyte salts, are non-flammable and have low raw material costs. However, they are not compatible with all electrode materials in LT applications. SO_2_-based inorganic liquid electrolytes have low electrical resistance, which helps to improve the Li^+^ transport rate at LT. They are suitable for a wide range of commercial electrode materials, have excellent cycling and multiplication performance, and are inherently non-flammable, providing high safety. SO_2_-based inorganic liquid electrolytes, however, have not yet been used on a large scale.

Considering the wide range of applications of LIBs in EVs, research on the application of high safety LIBs in cryogenic environments is complex and important. We need to consider the composition of different types of electrolytes, as well as consider the production cost, difficulty of the manufacturing process, and the effect of actual low-temperature applications. We believe that through the study of different types of LIB electrolytes at LT, high-safety LIBs will become the mainstream of new energy applications in cryogenic environments in the future. By overcoming the challenges of extreme temperatures, we can further promote EVs as the sustainable transportation of the future.

## Figures and Tables

**Table 1:** The performance of LIBs with different electrolytes.

Type	Composites	Ionic Conductivity (S cm^−1^)/ Measuring Temperature (°C)	Initial Capacity (mAh g^−1^)/ Capacity Rentation (%)/ Current Density/ Cycle Number/ Measuring Temperature (°C)	Reference
DEEs	MSA:LiFSI 3:1	>1.0 × 10^−4^/−30 °C	-	[66]
	DMMSA:LiFSI 4:1	>1.0 × 10^−4^/−30 °C		
	LiPF_6_-DEE	7.78 × 10^−4^/0 °C	-	[67]
	LiTFSI-DEE	8.26 × 10^−4^/0 °C		
	D-DES	1.0 × 10^−3^/RT		[68]
	LiMn_2_O_4_//LT-1//LiTi_2_ (PO_4_)_3_	-	~60 mAh g^−1^/97.5%/0.1 A g^−1^/20 cyc/−20 °C	[69]
			~95 mAh g^−1^/98%/0.1 A g^−1^/20 cyc/−10 °C	
			~60 mAh g^−1^/97.5/0.1 A g^−1^/20 cyc/−20 °C	
	LMO//CP-131//LTO	-	120 mAh g^−1^/83.3%/1 C/10 cyc/0 °C	[72]
			80 mAh g^−1^/95%/1 C/10 cyc/−10 °C	
			40 mAh g^−1^/97%/1 C/10 cyc/−20 °C	
SSE-ISEs	LSiGePSBrO	9 × 10^−3^/−10 °C	-	[82]
	NCM622//Li_5_._5_PS_4_._5_Cl_1_._5_//Li	-/−20 °C	~57 mAh g^−1^/88.4%/1 C/100 cyc/−20 °C	[79]
SSE-SPEs	Li//DOL/Al(OTF)_3_/LiTFSI//Li	3.26 × 10^−4^/0 °C	stable cycle of 710 h at a rate of 0.2 mA cm^−2^ with 0 °C	[88]
	DOL/FEC/Al(OTF)_3_/LiTFSI(FEC = 5 vol.%)	-	-	
	LFP//Poly-DOL-10//Li	2.4 × 10^−5^/−60 °C	~70 mAh g^−1^/104.7%/0.2 C/100 cyc/−20 °C	
		9.86 × 10^−4^/0 °C	~112 mAh g^−1^/111.2%/0.2 C/400 cyc/0 °C	
	DOL/FEC/Al(OTF)_3_/LiTFSI(FEC = 15 vol.%)	1.56 × 10^−3^/0 °C	-	
	Li//TXE-FDMA//NCM811	-/0 °C	~187 mAh g^−1^/99.7%/0.1 C/10 cyc/0 °C	[93]
		2.2 × 10^−4^/−20 °C	~130 mAh g^−1^/99.1%/0.1 C/200 cyc/−20°C	
	Li//TXE-FDMA//LFP	-/−20 °C	~83 mAh g^−1^/114.4%/0.085 C/350 cyc/−20°C	
	LFP//Li-PEG@NUST-21//Li	7.55 × 10^−7^/−40 °C	~110 mAh g^−1^/113.2%/0.1 C/82 cyc/10 °C	[97]
		5.41 × 10^−5^/0 °C		
	LFP//Li-PEG@NUST-22//Li	4.63 × 10^−7^/−40 °C	~125 mAh g^−1^/84.64%/0.1 C/94 cyc/10 °C	
		3.83 × 10^−5^/0 °C		
	LFP//Li-PEG@NUST-23//Li	9.74 × 10^−7^/−40 °C	~125 mAh g^−1^/106.9%/0.1 C/94 cyc/10 °C	
		7.10 × 10^−5^/0 °C		
	Li//SI10-05-70%PC//NCM811	6 × 10^−4^/20 °C	109 mAh g^−1^/90%/0.5 C/500 cyc/0 °C	[98]
		>1 × 10^−4^/−30 °C		
	Li//PTFSI-10/5-PC//NMC622	2.15 × 10^−4^/20 °C	~75 mAh g^−1^/80%/0.5 C/300 cyc/0 °C	[99]
		8.98 × 10^−5^/0 °C		
	LFP//LiQSSE//Li	3.9 × 10^−5^/−20 °C	~53 mAh g^−1^/93%/0.5 C/90 cyc/−10 °C	[100]
SSE-CESs	LFP//PPPL-10//Li	0.4 × 10^−3^/25 °C	~109 mAh g^−1^/99.9%/0.2 C/100 cyc/0 °C	[101]
	NCM811//PVLN-15//Li	-	~142 mAh g^−1^/118.3%/0.1 C/100 cyc/−20 °C	[102]
	ZnO-PEO	>1 × 10^−4^/0 °C	-	[103]
	PEO-HPyr	>1 × 10^−6^/−20 °C	-	[104]
	LTO//CSE//Li	>1 × 10^−4^/−10 °C	~160 mAh g^−1^/94.38%/0.4 C/50 cyc/−10 °C	[105]
SSE-PCEs	LiPBu_4_TFSI@TPB-DMTP-COF	2.1 × 10^−2^/−30 °C	-	[109]
	LCO//PPCE-3//LTO	6 × 10^−4^/−20 °C	128 mAh g^−1^/-/15mA g^−1^/1 cyc/−5 °C	[110]
			120 mAh g^−1^/-/30mA g^−1^/1 cyc/−5 °C	
			98 mAh g^−1^/-/75mA g^−1^/1 cyc/−5 °C	
			73 mAh g^−1^/-/150 mA g^−1^/1 cyc/−5 °C	
			46 mAh g^−1^/-/300 mA g^−1^/1 cyc/−5 °C	
ILs	LiTFSI-PYR_13_TFSI-PYR_13_FSI	1 × 10^−3^/−20 °C	-	[118]
	Li//LiFSI^−^[PP_13_]^+^[FSI]^−^HFE//graphite	-/RT	~230 mAh g^−1^/82.61%/3 C/300 cyc/RT	[119]
		-/−10 °C	352 mAh g^−1^/-/0.05 C/1 cyc/−10 °C	
		-/−20 °C	325 mAh g^−1^/-/0.05 C/1 cyc/−20 °C	
		6 × 10^−4^/−30 °C	245 mAh g^−1^/-/0.05 C/1 cyc/−30 °C	
	Li//[LiFSI]_1_[EmimFSI]_2_[dFBn]_2_//Li	1.67 × 10^−3^/−20 °C	stable cycle of 1000 h at a rate of 0.25 mA cm^−2^ with −20 °C	[126]
	Li//[LiFSI]_1_[EmimFSI]_2_[dFBn]_2_//NCA		156 mAh g^−1^/85.9%/1 C/500 cyc/−20 °C	
	[LiFSI]_1_[EmimFSI]_2_	5 × 10^−4^/−20 °C	-	
	Li//[LiFSI]_1_[EmimFSI]_2_[dFBn]_2_//NCM811	-/RT	192 mAh g^−1^/93%/1 C/500 cyc/RT	[128]
IEs	LiMn_2_O_4_//LiPTFSI:LiOTf(15 m:5 m)//Li	-	110 mAh g^−1^/-/1 C/1 cyc/0 °C	[130]
	LiMn_2_O_4_//LiPTFSI:LiOTf(15 m:5 m)//Li		100 mAh g^−1^/-/1 C/1 cyc/0 °C	
	LiMn_2_O_4_//BSiS-A_0_._5_//LTO	1.34 × 10^−3^/0 °C	~116 mAh g^−1^/95%/1 C/120 cyc/0 °C	[131]
		6.3 × 10^−4^/−20 °C		
	Li//LiAlCl_4_·3SO_2_//LFP	2.377 × 10^−2^/RT	120 mAh g^−1^/93.7%/5 C/100cyc/RT	[142]
		-/0 °C	110 mAh g^−1^/104%/2 C/100cyc/0 °C	
	Li//LiAlCl_4_·3SO_2_//LFP	6.54 × 10^−3^/−20 °C	~165 mAhg^−1^/95%/0.5 C/95 cyc/RT	[143]
		11.04 × 10^−3^/−10 °C		
		27.51 × 10^−3^/0 °C		
	Li//LiAlCl_4_·3SO_2_//LTO	-/RT	151 mAh g^−1^/91.23%/5 C/500 cyc/RT	

## Data Availability

Data are contained within the article.
